# Imaging mass spectrometry and MS/MS molecular networking reveals chemical interactions among cuticular bacteria and pathogenic fungi associated with fungus-growing ants

**DOI:** 10.1038/s41598-017-05515-6

**Published:** 2017-07-17

**Authors:** Cristopher A. Boya P., Hermógenes Fernández-Marín, Luis C. Mejía, Carmenza Spadafora, Pieter C. Dorrestein, Marcelino Gutiérrez

**Affiliations:** 1 Centro de Biodiversidad y Descubrimiento de Drogas, Instituto de Investigaciones Científicas y Servicios de Alta Tecnología (INDICASAT AIP), Panamá, Apartado 0843-01103 Republic of Panama; 2 Department of Biotechnology, Acharya Nagarjuna University, Guntur, Nagarjuna Nagar, 522 510 India; 3 Centro de Biología Celular y Molecular de Enfermedades, INDICASAT AIP, Panamá, Apartado 0843-01103 Republic of Panama; 4 Collaborative Mass Spectrometry Innovation Center, Skaggs School of Pharmacy and Pharmaceutical Sciences, University of California at San Diego, San Diego, California 92093 United States; 5 Department of Pharmacology, University of California at San Diego, San Diego, California 92093 United States

## Abstract

The fungus-growing ant-microbe symbiosis is an ideal system to study chemistry-based microbial interactions due to the wealth of microbial interactions described, and the lack of information on the molecules involved therein. In this study, we employed a combination of MALDI imaging mass spectrometry (MALDI-IMS) and MS/MS molecular networking to study chemistry-based microbial interactions in this system. MALDI IMS was used to visualize the distribution of antimicrobials at the inhibition zone between bacteria associated to the ant *Acromyrmex echinatior* and the fungal pathogen *Escovopsis* sp. MS/MS molecular networking was used for the dereplication of compounds found at the inhibition zones. We identified the antibiotics actinomycins D, X2 and X_0β_, produced by the bacterium *Streptomyces* CBR38; and the macrolides elaiophylin, efomycin A and efomycin G, produced by the bacterium *Streptomyces* CBR53.These metabolites were found at the inhibition zones using MALDI IMS and were identified using MS/MS molecular networking. Additionally, three shearinines D, F, and J produced by the fungal pathogen *Escovopsis* TZ49 were detected. This is the first report of elaiophylins, actinomycin X_0β_ and shearinines in the fungus-growing ant symbiotic system. These results suggest a secondary prophylactic use of these antibiotics by *A*. *echinatior* because of their permanent production by the bacteria.

## Introduction

Symbioses are associations between different organisms which involve a high degree of dependence and coexistence and are broadly distributed in nature^1^. Such associations can be found in any ecosystem including most habitat types on earth, and the effects that one or more members of a symbiotic relationship have on each other can affect the system as a whole^2–5^. Living in symbiosis is an evolutionary strategy enabling organisms to interact. However, there are few examples that explain the maintenance, mechanisms, and survivorship of symbiosis that involve more than two symbionts^6–8^. To improve our understanding of the mechanisms that regulate the communication and interactions among organisms living in symbiosis, studies need to focus on exploring the nature and diversity of the chemical compounds produced by the organisms interacting in a symbiotic system^3, 9–12^.

The most complex case of symbiosis may be where an obligatory mutualism occurs in which each symbiotic partner is influenced by their co-evolutionary relationship^13^. The fungus-growing ants are part of a complex symbiosis between the ants and their microbes, where the attines maintain an obligatory mutualism with a basidiomycete fungus (cultivars) in the tribe Leucocoprineae, which is grown as a unique food source for the brood^14–16^. At least three other microbes have evolved in this system including: 1) antibiotic-producing bacteria in the order Actinomycetales, which are producers of compounds that inhibit the growth of fungal ant pathogens^17^; 2) a specialized micro-parasite of the cultivar in the genus *Escovopsis* ^18^; and 3) the least studied symbionts are black yeasts, that are antagonists of the antibiotic-producing bacteria^19, 20^.

Uncontrolled infections of the cultivar by pathogen *Escovopsis* sp. can cause the death of an entire ant colony. Therefore ants employ a diversity of strategies for maintaining a healthy colony including hygienic behavior, social immunity, waste management, and the use of antimicrobial compounds^21, 22^. The fungus-growing ant symbiotic system has a diversity of small molecules that potentially mediate interactions among the microbial members of this symbiosis^23–27^, and may additionally be a rich source of compounds for drug discovery programs^23^.

Interactions among antibiotic-producing bacteria (genus *Pseudonocardia* and *Streptomyces*) and the mutualistic basidiomycete and *Escovopsis* have been the subject of several studies. Such studies have shown that some ant-associated bacteria are able to produce compounds that inhibit the growth of *Escovopsis* and other entomopathogens, with and without affecting the cultivar^28–31^. Despite the fact that many of these antagonistic interactions have been described for the fungus growing ant system, in most cases the chemicals responsible for the inhibitory effects remains unknown. Previous work on the natural product chemistry of the bacteria associated with attine ants show that some bacterial metabolites are involved in the inhibition of *Escovopsis*. The few natural products that have been elucidated so far for the system include: dentigerumycin^24^; five angucycline antibiotics (pseudonocardones A–C, 6-deoxy-8-O-methylrabelomycin and X-14881 E)^23^, the antifungals (candicidin D, actinomycins D and X, valinomycin, antimycin A1-A4 and a several nystatin-like metabolites)^26, 32–34^; two antimycin antibiotics (urauchimycins A and B)^23, 35^ (Table 1).

**Table 1:** Metabolites described for antibiotic producing bacteria associated to the fungus-growing ants.

Fungus growing ants species	Bacteria	Bacterial activity	Compounds isolated	Compounds activity	Reference
*Apterostigma dentigerum*	*Pseudonocardia* spp.	*Escovopsis* sp.	dentigerumycin	*Escovopsis* sp.	24
	*Pseudonocardia* sp.	Not reported	selvamicin	*Candida albicans*, *Saccharomyces cerevisiae*, *Aspergillus fumigatus*, and *Trichoderma harzianum*	33
	*Pseudonocardia* sp. EC080529-01	Not reported	6-deoxy-8-O-methylrabelomycin	*Bacillus subtilis* 3610 and *Plasmodium berghei*	23
			X-14881 E (8-O-methyltetrangulol)		
			pseudonocardones A	Inactive for test organism	
			pseudonocardones B		
			pseudonocardones C		
*A*. *octospinosus*	*Pseudonocardia* sp.	*Candida albicans* and *E. weberi*	nystatin P1	Not tested	26
	*Streptomyces* sp.	*E*. *weberi*	candicidin D	*E*. *weberi*	26, 27, 32, 34
	*Streptomyces* sp. *Streptomyces* sp. Av25_2	Not reported	actinomycin D	*B*. *subtilis*	
*Acromyrmex* sp.	*Streptomyces* sp. Av25_2	Not reported	actinomycin X2	*B*. *subtilis*	34
	*Streptomyces* sp. Av25_3	Not reported	valinomycin	*B*. *subtilis*	
	*Streptomyces* sp. Ao10	*E*. *weberi*	antimycin A1	*E*. *weberi*	
			antimycin A2		
			antimycin A3		
			antimycin A3		
*Trachymyrmex* sp.	*Streptomyces* sp. TD025.	Five *Candida* species	urauchimycin A	Four *Candida* species	35
			urauchimycin B	Six *Candida* species	

The table summarizes compounds that are produced by the bacteria associated with fungus growing ant’s species pointing out the activity against the fungal pathogen, *Escovopsis*.

The lack of information available on the chemical nature of the interactions between members of the attine ant-microbes system provides an opportunity to explore the production of interaction-mediator compounds, their metabolic pathways and their ecological role in the symbiosis. The use of techniques such as MALDI imaging mass spectrometry, MS-based molecular networking^36–40^, and metagenomics approaches^41^ can provide new insights into how microbial communities associated with attine ants interact at the molecular level.

Herein we used MALDI-IMS^36, 37, 40^ and MS/MS-based molecular networking^38, 39, 41^ to study antagonistic interactions between actinobacteria in the genus *Streptomyces*, isolated from the ant *Acromyrmex echinatior*, against strains of *Escovopsis* isolated from the fungal garden of *A*. *echinatior* and *Trachymyrmex zeteki* nests. Using these techniques we found a series of antimicrobial metabolites produced by *Streptomyces* CBR53 and *Streptomyces* CBR38. The conditions under they were produced, and their antimicrobial properties in the fungus-growing ants symbiotic system were assessed.

## Results

### DNA sequence identification

Bacterial strains CBR53 and CBR38 isolated from the propleura of *A*. *echinatior* were both identified as *Streptomyces* sp. based on the analysis of their partial 16S rRNA gene sequences. 16S rRNA gene sequences were deposited in Gene Bank with accession numbers KM096868 and KM096867 for CBR53 and CBR38, respectively. Fungal pathogen strains ACRO424 and TZ49 were identified as *Escovopsis* sp. based on the partial sequence of the internal transcribed spacers (ITS), sequences were deposited in Gene Bank under the accession numbers KM096865 and KM096866.

### Identification of metabolites using MALDI IMS and molecular networking of *Streptomyces* CBR53 and *Escovopsis* TZ49 extracts

*Streptomyces* CBR53 showed an inhibition zone diameter of 3.47 ± 0.51 mm against *Escovopsis* TZ49. *Streptomyces* CBR53 metabolites not only affected the side that was head-on with *Escovopsis*, they additionally exert a negative effect on the opposite side of the radial growth of *Escovopsis*, reducing from normal grow of 17.19 ± 2.67 mm to 4.33 ± 0.14 mm in the presence of the bacteria.

**Figure 1:**
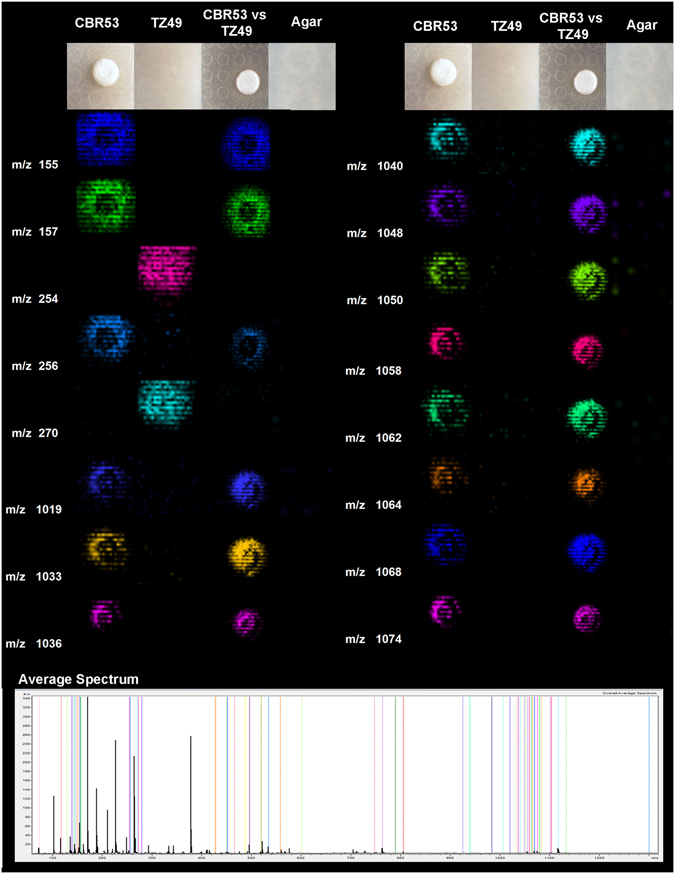
MALDI-TOF imaging mass spectrometry of microbial interactions between *Streptomyces* CBR53 and pathogenic fungi *Escovopsis* TZ49. Representative ions observed for the microbial interaction are presented as columns, first column show mass to charge ratio, second to fifth columns display false-colored images of the spatial distribution of the ions observed around: *Streptomyces* sp. (CBR53); *Escovopsis* sp. ﻿(TZ49)﻿;﻿ *Streptomyces* sp. vs *Escovopsis* sp. (CBR53vsTZ49); and SFM Agar. Average mass spectrum highlighting all the selected ions is shown at the bottom.

Using MALDI IMS we evaluated the pairwise interaction between *Streptomyces* sp. CBR53 and *Escovopsis* sp. TZ49, the results captured the distribution of several ions (metabolites) produced by the bacteria *(m/z* 519, 534, 924, 1033, 1048, 1062 and 1064, among others) and few from the fungi (*m/z* 254 and 270) (Fig. 1, Supplementary Figs S1 and S2). A detailed examination of the region between m/z 534 to *m/z* 1080 of the MALDI IMS experiments, showed that some compounds have similar spatial distribution around the *Streptomyces* sp. CBR53 growth in both inoculum procedures: single *Streptomyces* sp. CBR53 (Fig. 1: CBR53) and *Streptomyces* sp. vs *Escovopsis* sp. (Fig. 1: CBR53vsTZ49); indicating a lack of influence of the fungi towards the bacteria regarding the production of these metabolites. Furthermore, *Escovopsis* sp. TZ49 metabolites, ions at *m/z* 254 and 270 (Fig. 1: TZ49), are completely absent in the pairwise match (Fig. 1: CBR53vsTZ49; Supplementary Figs S1 and S2) likely because the presence of the bacterial metabolites.

The chemical profile of the methanol extract of *Streptomyces* sp. CBR53 analyzed by high resolution ESI-TOFMS showed some ions at *m/z* 1033.5589, 1047.5779, 1061.5799, 1079.6032 that match with the ions visualized in the MALDI IMS experiment (Supplementary Figure S2).

**Figure 2:**
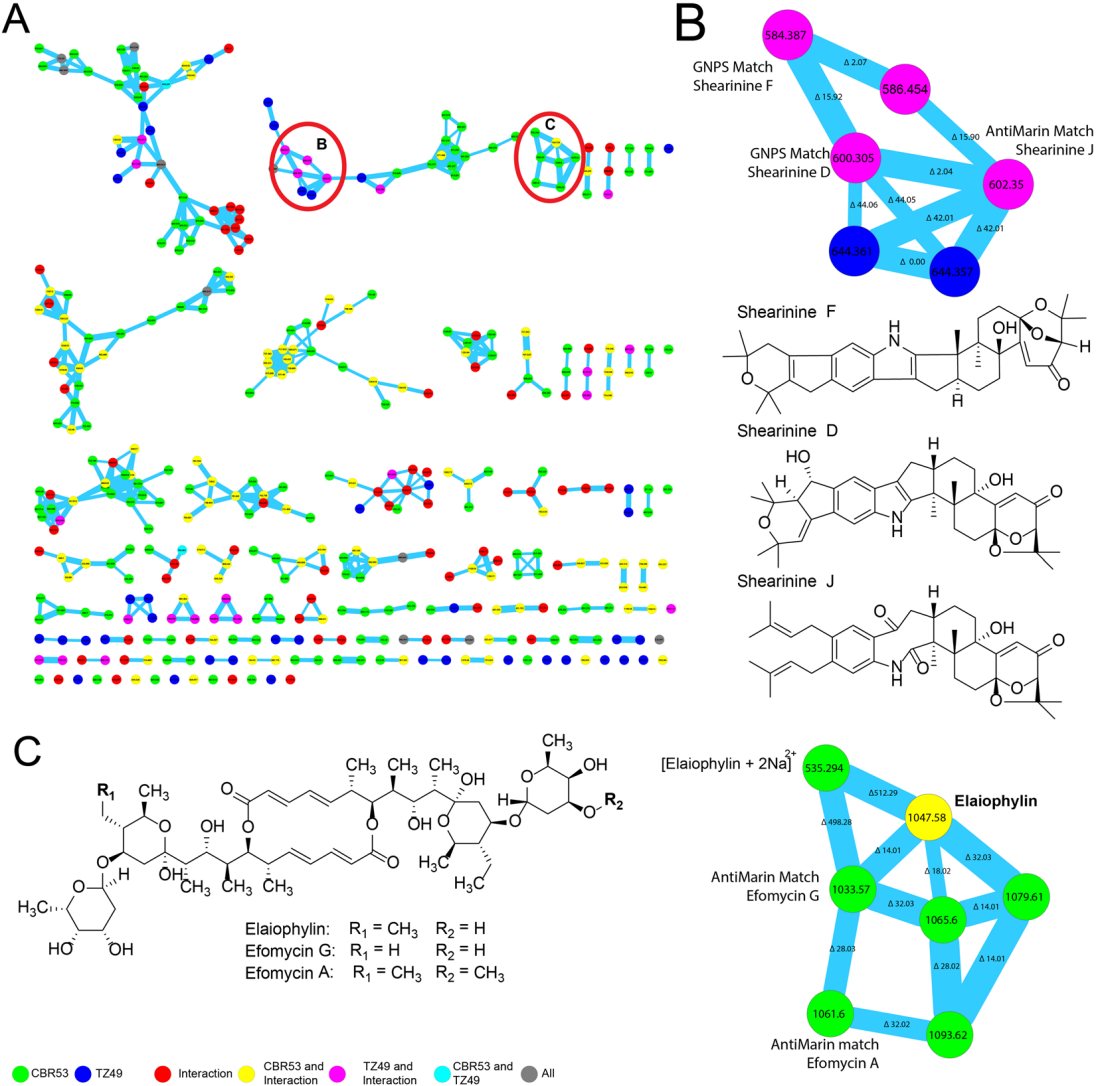
Molecular network from the extract of the bacterium *Streptomyces* CBR53 with the identification of the elaiophylin macrolide. (**A**) Molecular network after filter blanks, colors indicate producers of the nodes: *Streptomyces* CBR53 green; *Escvopsis* TZ49 blue, *Streptomyces* sp. vs *Escovopsis* sp. (CBR53 vs TZ49) red. Nodes found in more than one producers are represented as combination of their colors: *Streptomyces* CBR53, *Streptomyces* sp. vs *Escovopsis* sp. (CBR53 vs TZ49) yellow; *Escovopsis* TZ49, *Streptomyces* sp. vs *Escovopsis* sp (CBR53 vs TZ49) pink; *Streptomyces* CBR53, *Escvopsis* TZ49 cian. (**B**) Enlarged cluster of shearinine derivatives indicating their location in the sub-network, chemical structures of shearinine D, shearinine F and shearinine J. (**C**) Chemical structures of elaiophylin, efomycin G and efomycin A macrolides dereplicated using GNPS and AntiMarin, enlarged cluster denoting their location in the cluster.

MS-guided fractionation of the methanol extract of *Streptomyces* sp. CBR53 was carried out using a reverse phase solid phase extraction cartridge (SPE-C18) followed by HPLC purification yielding compound 1. Compound 1 (*m/z* 1047.5779 [M + Na]^+^) was identified as elaiophylin (Fig. 2C) on the basis of the comparison of its spectral data (Supplementary Figs S3, S4, S5 and Table S1) with spectroscopic data from the literature^42–47^.

To characterize additional compounds we employed the HPLC ESI-Q-TOF-HRMS data of the methanol extract of: *Streptomyces* sp. CBR53, *Streptomyces* sp. vs *Escovopsis* sp. (CBR53vsTZ49) and *Escvopsis* sp. TZ49, to create a molecular network using the online workflow at GNPS (http://gnps.ucsd.edu/ProteoSAFe/status.jsp?task=013afe575fb7438a96c9ab60da167f98). As a result, a molecular network (Fig. 2A) consisting of three hundred seventy seven (377) nodes, clustered in seventy (70) spectral families and 29 individual nodes was obtained, after filtering nodes from background (matrix, agar and solvent). A search of each MS/MS spectrum against GNPS’s spectral libraries resulted in the dereplication of three compounds including elaiophylin, shearinine D and shearinine F.

Elaiophylin was found in a seven (7) nodes cluster in which all ions were produced by *Streptomyces* CBR53 in single culture and interaction with *Escovopsis* TZ49 (Fig. 2C and Supplementary Fig. S5); dereplication of these nodes using the AntiMarin database resulted in the identification of additional metabolites including efomycin G^43, 48^ and efomycin A^48^, along with the elaiophylin double-charged adduct [M + 2Na]^2+^ (Fig. 2C, Supplementary Figs S6 and S7).

HRESIQTOF MS^2^ analysis of efomycin G at m/z 1033.5742 [M + Na]^+^ (calculated for C_54_H_88_O_18_Na, 1033.5706) showed three mayor fragments at m/z: 411.1819, 715.3734 and 729.3860 which correspond to the aglycone and the cleavage of the nonsymmetrical groups between carbons 9-10 and 9″-10″ respectively, probably following a retro-heteroene rearrangement^49, 50^ (Supplementary Fig. S6). In a similar way efomycin A showed a fragmentation pattern consisting of three mayor fragments at m/z: 411.1800 which correspond to the aglycone; and the ions at *m/z* 743.3699 and 729.3763, which indicate a cleavage of the nonsymmetrical groups between carbons 9–10 and 9′′–10′′ (Supplementary Fig. S7).

We were unable to dereplicate three nodes of the elaiophylin cluster at *m/z* 1065.60, 1079.61 and 1093.62, with mass variations of 18, 32 and 46 Daltons compared to elaiophylin. These mass shifts suggests the presence of an extra atom of oxygen for node *m/z* 1065.60, two extra oxygen atoms for *m/z* 1079.61 and two extra oxygen plus a methyl group for *m/z* 1093.62 (Supplementary Fig. S8). These compounds are very likely new members of the elaiophylin family and their isolation and structural determination are needed in order to identify them.

Additionally a cluster of twenty three (23) nodes composed of metabolites produced by the *Escovopsis* sp. TZ49 in single culture and in the interaction with *Streptomyces* CBR53 was observed (Fig. 2B). Manual comparison of the MS/MS spectra of the nodes found in this cluster against the GNPS library resulted in the dereplication of shearinines D and F^51^ (Supplementary Figs S9 and S10). One of the nodes of the shearinines cluster at m/z 602.35200 didn’t match with any compound at the GNPS library, however it matched with shearinine J at the AntiMarin database.

The antimicrobial activity of elaiophylin was evaluated against several bacteria and fungi including *Escovopsis* (TZ49 and ACRO424), *Aspergillus fumigatus*, *Candida albicans*, *Staphylococcus aureus* subsp. *aureus* and *Bacillus*. *subtilis* subsp. *subtilis*. Elaiophylin did not show activity against the *Escovopsis* and *Aspergillus* strains assessed in this study, however antibacterial activity was observed against *S*. *aureus subsp*. *aureus* showing a minimum inhibitory concentration (MIC) of 13.7 µM; also against *B*. *subtilis subsp*. *subtilis* with a MIC value of 17.0 µM; and mild activity was observed against *C*. *albicans* with a MIC value of 102.2 µM.

Elaiophylin was also evaluated against parasites (IC_50_ value of 0.68 µM against the *Plasmodium falciparum* strain HB3, IC_50_ of 0.946 µM against *Trypanosoma cruzi*), and human breast cancer (IC_50_ value of 0.67 µM for the MCF-7 cell line).

### Identification of metabolites using MALDI IMS and molecular networking of extracts from *Streptomyces* CBR38 and *Escovopsis* ACRO424

**Figure 3:**
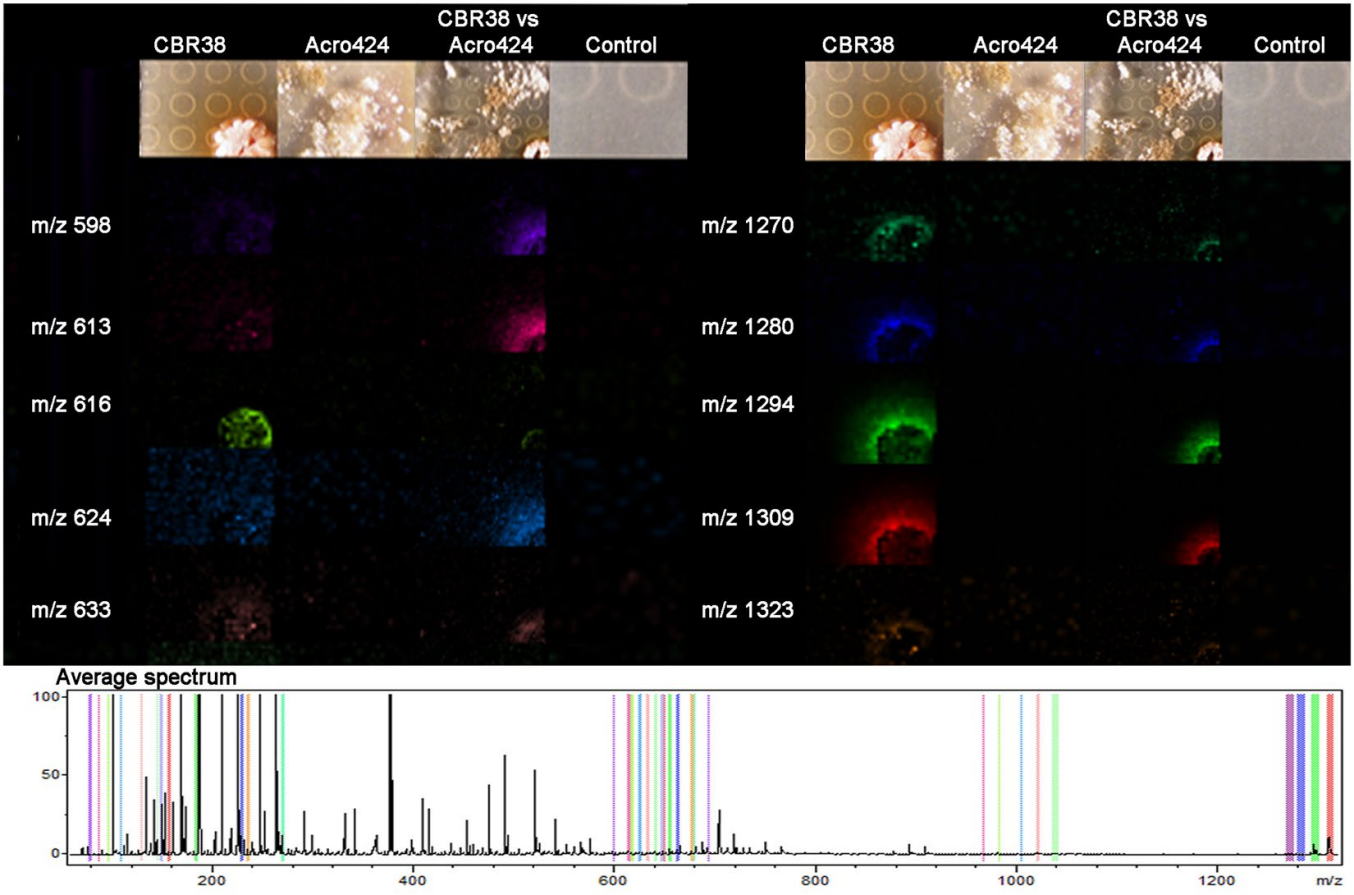
MALDI-TOF imaging mass spectrometry experiment of the bacterium *Streptomyces* CBR38 vs *Escovopsis* ACRO424. Selected ions observed in the microbial interaction are presented as columns, first column show mass to charge ratio, second to fifth columns display false-colored images of the spatial distribution of the ions detected around: *Streptomyces* CBR38; *Escovopsis* ACRO424; *Streptomyces* sp. vs *Escovopsis* sp.(CBR38 vs ACRO424); and SFM Agar. Average mass spectrum highlighting all the selected ions is show at the bottom.

A pairwise interaction experiment among *Streptomyces* sp. CBR38 and *Escovopsis* ACRO424, both isolated from *Acromyrmex echinatior* nest, was carried out with the aim of understanding the microbial interaction between beneficial and antagonist microbes of this higher attine group. Co-culture in SFM media of *Streptomyces* sp. CBR38 and *Escovopsis* sp. ACRO424 for 10 days resulted in a clear inhibition of the fungi displaying an inhibition zone diameter of 4.49 ± 0.15 mm. MALDI IMS was used to elucidate the spatial spreading of metabolites in the interaction. The results showed several ions surrounding the bacterial growing, for instance *m/z* 624, 633, 1270, 1294 and 1309 (Fig. 3 and Supplementary Fig. S11). These metabolites were detected in the single culture of *Streptomyces* sp. CBR38 (Fig. 3: CBR38) and during the pairwise experiment of *Streptomyces* sp. against *Escovopsis* sp. (Fig. 3: CBR38 vs ACRO424; Supplementary Fig. S11). Only one metabolite was assigned in association with the fungi at *m/z* 270 (Fig. S11).

The chemical profile of the ethyl acetate extract of *Streptomyces* sp. (CBR38) carried out by high resolution IT-FTICRMS showed ions at *m/z* 1255.6578, 1269.6329 and 1271.6213 which corresponded to the [M + H]^+^ adducts of the ions visualized in the MALDI IMS (1294 [M + H + K]^+^, 1309 [M + H + K]^+^ and 1270 Fig. 3) experiments. Dereplication of these ions using the AntiMarin database matched with actinomycin D and actinomycin X2 and actinomycin X_0β_^52^, respectively.

**Figure 4:**
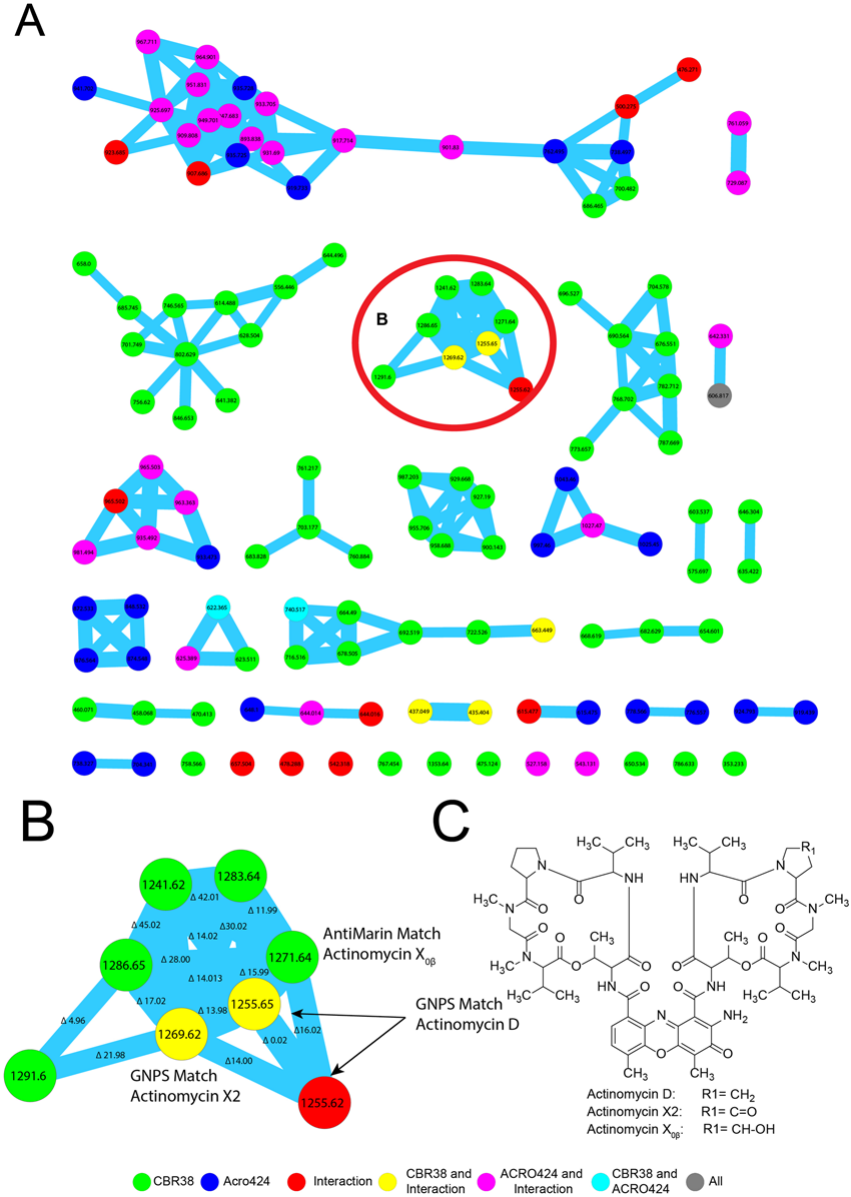
Molecular network from *Streptomyces* CBR38 and *Escovopsis* Acro424 with the identification of the actinomycins. (**A**) Molecular network after filter blank, colors indicate producers of the nodes: *Streptomyces* CBR38 green; *Escvopsis* ACRO424 blue, *Streptomyces* sp. vs *Escovopsis* sp (CBR38 vs ACRO424) red. Nodes found in more than one producers are represented as combination of their colors: *Streptomyces* CBR38, *Streptomyces* sp. vs *Escovopsis* CBR38 vs ACRO424 yellow; *Escvopsis* ACRO424, *Streptomyces* sp. vs *Escovopsis* sp (CBR38 vs ACRO424) pink; *Streptomyces* CBR38, *Escvopsis* ACRO424 cian. (**B**) Enlarged cluster of actinomycin D, actinomycin X2 and actinomycin X_0β_. (**C**) Chemical structures of actinomycin D, actinomycin X2 and actinomycin X_0β_.

In order to confirm the identity of these compounds we used the HRESI-Q-TOF MS/MS data of the methanol extract of: *Streptomyces* sp. CBR38, *Streptomyces* sp. CBR38 vs *Escovopsis* sp. ACRO424 interaction and *Escovopsis* ACRO424, in order to create a molecular network using the online workflow at GNPS (http://gnps.ucsd.edu/ProteoSAFe/status.jsp?task=7043d3de65554f7aa127d343191abf6c). As a result we obtained a molecular network consisting of one hundred twenty five (125) nodes, clustered in twenty three molecular families and twelve individual nodes, after filtering nodes from matrix (agar and solvent) (Fig. 4A). Analysis of this data showed a cluster of eight nodes (Fig. 4B) containing the ions *m/z* 1255.6400, 1269.6200 and 1271.6415, among others. Search of each MS^2^ spectrum of these ions against GNPS’s spectral libraries resulted in the dereplication of actinomycin D, actinomycin X2, and actinomycin X_0β_ (Fig. 4C). Actinomycin D at *m/z* 1255.6578, showed a cosine score of 0.90 and 41 shared peaks. Identification of the compound was supported by direct comparison of the MS^2^ spectra of actinomycin D (MS/MS from *Streptomyces* sp. CBR38) with a standard of actinomycin D from GNPS by mirror plot using mMass software^53^ (Supplementary Fig. S12). The ion m/z 1269.6329 was identified as actinomycin X2 with a cosine score of 0.91 and 49 shared peaks. Direct comparison of MS/MS spectra from GNPS standard of actinomycin X2 by mirror plot confirmed the identity of this compound (Supplementary Fig. S13). The ion m/z 1271.6415 was identified as Actinomycin X_0β_ by direct comparison with MS/MS spectra from GNPS standard of actinomycin X_0β_ (Supplementary Fig. S14). This GNPS-based analysis supported the presence of actinomycins in our bacterial extracts. The dereplication of other ions present in the cluster using AntiMarin Database matched with other members of the vast family of actinomycins, however we were unable to identify all of them because GNPS’s libraries contains only some actinomycin analogues.

## Discussion

Microbes in the attine ant system use their biosynthetic machinery to produce metabolites dedicated to interact with the host, and also with other microbial symbionts in the system. However the specific purposes for which each of these metabolites are primarily produced remains unknown. Through microbial interactions using metabolites, microbes likely: get access to nutrients and also obtain protection and adaptation to different ecological niches^54^. Attine ants maintain a complex symbiotic system in which microbial interactions play a key role to keep the equilibrium in the fitness of the community, involving host-symbiont relationship and symbiont-symbiont microbial interactions.

Herein a combination of MALDI imaging mass spectrometry (MALDI-IMS) and mass spectrometry-based molecular networking was used to study microbial interactions in the attine ants system, specifically between actinobacteria and *Escovopsis*. In this work we report the identification of three molecular families associated to microbes in the attine ants system: the elaiophylins from *Streptomyces* sp. CBR53, the actinomycins from *Streptomyces* sp. CBR38 and a group of triterpenic indole alkaloids named shearinines from *Escovopsis* sp.TZ49. Elaiophylin macrolide and its analogues are well known antibiotics produced by several *Streptomyces* strains, these macrolides are classified as entities of biological interest^55^ presenting a wide range of antibacterial, antifungal, antiinflamatory, enzyme inhibition and anticancer activities^43, 46, 48, 56–59^. The actinomycins present a wide range of antifungal^34, 60–64^ antibacterial^65^ and anticancer^66, 67^ properties. On the other hand, the shearinines are alkaloids previously reported from fungi in the genus *Eupenicillium* and *Penicillium*. These compounds has been reported for anti-leukemia activity^68^, and also exhibit *in vitro* blocking activity on large-conductance calcium-activated potassium channels^51^, antiinsectan^69^ and anti-*Candida* activities^70^.

Based on our results and the known biological activities of the compounds reported in this work we suggest that the actinomycins D, X2 and X_0β_ found in the *Streptomyces* sp. CBR38, are involved in the fungistatic action of this bacterium against *Escovopsis*, due to they were found distributed around the inhibition zone, observed in the MALDI IMS experiments (Fig. 3 and Supplementary Fig. S11). On the other hand the elaiophylin and its analogues, found in *Streptomyces* sp. CBR53, are widely known for their antibacterial activity; hence we suggest they are more likely involved in the control of bacteria present in the nest of *Acromyrmex* ants.

Our data suggest that ant colonies require constant production of these antimicrobial compounds in order to control pathogens present in the nest; since *Escovopsis* apparently do not exert a stimulus over the bacteria as shown in the MALDI IMS experiments for both bacterial strains (Figs. 1, 3, Supplementary Figs S1, S2 and S11). The molecular network studies also show that some metabolites belong to *Streptomyces* and other to *Escovopsis* (Figs 2 and 4). However it is unknown if the ants could influence the production of this bacterial metabolites during infections of their nest. Currie *et al*. demonstrated that an increase in the concentration of actinobacteria associated to ants occurs after the inoculation of *Escovopsis* in the nest. They inferred this increase of actinomycetes bacteria as an strategy to control *Escovopsis* infection and proliferation^13^. Based on our results, we support the hypothesis of Currie *et al*. suggesting that an increase in the concentration of bacteria would result in an increase in the concentration of secreted compounds. However, there is no evidence of the minimal concentrations of bacteria required to control *Escovopsis* using these compounds, neither if there is an inhibition of the production of compounds by the bacteria once an optimum concentration is reached.

MALDI IMS experiments allowed us to detect few metabolites from *Escovopsis*, this result could be explained because of the sample pre-treatment, in which the excessive amount of mycelia produced by *Escovopsis* was removed and only a minimum amount is present in the target plate before applications of the ionization matrix^71^. This procedure may also disturb the agar surface affecting the shape or thickness of the agar which result in a distortion of the mass spectra, making the manual analysis of the data more challenging as it introduces small mass shifts (0.5 to 1 Da) to the parent mass. However, the HRESI-Q-TOF MS/MS data of the methanol extract of *Escovopsis* sp. TZ49 allowed us to identify the shearinines D, F and J. The identification of these compounds support the computational predictions of terpene secondary metabolites in the genome *Escovopsis weberi* ^72^.

Earlier studies (Table 1) consistently demonstrate that ants employ a community of bacteria to synthetize a variety of compounds for the control of *Escovopsis* and entomopathogens. Our results also report a high number of compounds produced by bacteria, established by the presence of ions observed in the MALDI IMS experiments.

In this work we describe the presence of three molecular families represented by the macrolide antibiotic elaiophylin isolated from *Streptomyces* CBR53; the actinomycins detected in *Streptomyces* CBR38 and the shearinines detected in *Escovopsis* TZ49 using IT-FTICRMS or HRESI-TOFMS combined with MS^2^ fragmentation experiments of the extracts. These metabolites were detected around the inhibition zones in the MALDI IMS experiments carried out for each bacteria and fungi. However more detailed studies must be conducted in order to characterize all the ions observed in the MALDI IMS experiments (Figs. 1, 3, Supplementary Figs S1, S2 and S11) and also all the ions observed in the molecular cluster of each bacteria generated by GNPS, twenty two unidentified clusters from *Streptomyces* CBR38-*Escovopsis* ACRO424 and sixty eight from *Streptomyces* CBR53- *Escovopsis* TZ49. Despite the fact that in this work we are characterizing the occurrence of three molecular families for these strains, MALDI IMS and GNPS tools suggests that the diversity of compounds associated with these bacteria and fungi is much greater than what it is reported in the scientific literature (Table 1).

We propose that the use of a diversity of chemicals is a strategy used by ants and their associated bacteria to reduce the chance of pathogens (opportunistic bacteria, fungi, yeast or the specialized pathogens *Escovopsis sp*.) for developing antibiotic resistance^73^. This hypothesis is based on the presence of multiple antibiotics, like the elaiophylins (and its related nodes not yet identified visualized as circular nodes in (Fig. 2C) or, actinomycin D, actinomycin X2 and actinomycin X_0β_(Fig. 4C), found in the inhibition zones produced by the *Streptomyces* strains^26, 60^.

A diversity of chemicals have been reported for the attine ants symbiotic system, including: i) the antibiotics produced by the actinobacteria and other bacteria^23, 24, 26, 27^ (ii) the metabolites secreted by the ants exocrine glands^21, 22, 73–75^ and (iii) the metabolites produced by cultivated fungus^76^. We postulate that the pressure exerted by the presence of this diversity of chemicals is used to avoid and control infections in the ant nest.

Our MALDI-IMS analysis and molecular networking of both bacteria revealed that elaiophylins and actinomycins were produced by the bacteria in the absence and presence of the pathogen *Escovopsis*, (Figs 1, 2, 3, 4, Supplementary Figs S1, S2 and S11), therefore we deduce that *Escovopsis* do not induce the production of these compounds. These effects correspondingly indicate a secondary prophylactic use of antibiotics in the nest of *A*. *echinatior* because of the permanent production of antibiotics by the bacteria. Similarly the production of shearinine alkaloids by *Escovopsis* TZ49 was not influenced by the precense of *Streptomyces* CBR53.

Understanding the chemistry and biology of the attine ant symbiotic system represent opportunities for new research lines that target the ecological importance of complex symbiotic systems, and also using those systems to explore the use of their chemical diversity in drug discovery. For instance, the fact that eliaophylin macrolide showed antibacterial, anticancer, antimalarial and antitrypanosomal activities in our assays clearly indicate the high potential for drug discovery of the natural products involved in this system.

Further studies including metagenomic, metatranscriptomic and metabolomic approaches would help to understand better the compositions of the microbial communities, their interactions and would give new insights on the influence of external factors in the regulation of their activities^77, 78^. It would also unlock substantial information of uncultured microbial diversity^79^. Furthermore, the chemical diversity should be assessed using comprehensive methodologies based on mass spectrometry which offers a valuable analysis of microbial proteomes and metabolomes^80^, potentially providing new molecules with therapeutic and biotechnological applications.

Other specific studies should be designed to clarify the fitness cost of antibiotics production and development of antibiotic resistance on the attine ant-microbe symbiotic system through direct and indirect interactions among all microorganisms^19, 20^ in order to understand how the microbial populations are organized and modulated^81^. Metabolic pathways also must be considered to evaluate if the pathways are silent, expressed only in the present of specific stimulus or under particular environmental conditions or activated permanently to produce metabolites^82–84^.

## Conclusions

Mass spectrometry-based techniques such as MALDI-IMS and MS/MS molecular networking revealed, for the first time, metabolites that are directly involved in antagonistic interactions between actinobacteria associated with *A*. *echinatior* against the pathogen *Escovopsis* and other microbes. The presence of actinomycin D and actinomycin X2, two metabolites previously reported from ant-associated bacteria is confirmed. Moreover we are reporting the occurrence of actinomycin X_0β_ in this system. Additionally we isolated and characterized the antibiotic elaiophylin and identified two analogs including efomycin A and efomycin G from *Streptomyces* CBR53. Also we detected the presence of the shearinines D, F and J for the first time associated with the fungal pathogen *Escovopsis* TZ49. Actinomycin X_0β_, the elaiophylins and the shearinines are reported for the first time in the attine ant symbiotic system.

Furthermore MALDI-IMS revealed that compounds were produced by the bacteria and fungi in the absence and presence of their antagonist microbes, suggesting that these microbes do not induce the production of these compounds on the others. These results likewise advocate a secondary prophylactic use of antimicrobials in the nest of *A*. *echinatior* because of their permanent production by the bacteria. Hence our work demonstrates that single bacterial strain produces multiple ions that are present on the inhibition zone. We also demonstrate the robustness of the molecular networking as an efficient strategy for dereplication to study microbial metabolites^38^.

Finally our results reveal the great potential of metabolites involved in microbial interactions of the attine ant symbiotic system to drug discovery, which demonstrated a variety of biological activities against microorganisms and parasites relevant to public health.

## Methods

### Study organisms

*Acromyrmex echinatior* and *Trachymyrmex zeteki* nests were collected^85, 86^ along Pipeline road, Gamboa, Panamá, in August of 2010, and in Boquerón, Chiriquí, Panamá, in December 2011. Ant nests, including the queen, workers, brood, and fungal gardens were placed in plastic containers^85, 86^, transported to the laboratory where they were fed and supplied with water twice times per week until studied.

Cuticular bacteria were obtained by directly scraping the propleura of *A*. *echinatior* ants over chitin agar media supplemented with cycloheximide and nystatin^87^. Purification of the bacterial strains was carried out by re-plating the different colonies on mannitol soy flour agar (SFM) until an axenic strain was obtained.

*Escovopsis* sp. fungus were isolated by collecting small pieces of the fungus garden (<0.5 cm diameter) of *A*. *echinatior* and *T*. *zeteki* and left onto a moist chamber upon fungus grows, presence of subglobose brown conidia^88^ confirm that the samples were *Escovopsis*. Purification was carried out by passages over potato dextrose agar (PDA)^89^.

### DNA extraction and sequencing

DNA of fungal isolates were extracted using the Gentra Puregene Tissue kit (Qiagen, Valencia CA) according to the manufacturer’s instructions and the ITS locus amplified and sequenced^90^. The 16S rDNA of bacteria were PCR amplified directly from pure culture of bacteria^91^. The 16S rDNA gene was sequenced using the BigDye Terminator sequencing kit v 3.1, in an ABI PRISM ®3100 Genetic Analyser (Applied Biosystems, Foster City, CA). DNA sequences were quality evaluated using the software Sequencher (Gene Codes Corporation, Ann Arbor, MI) and compared to the nucleotide database of the National Center for Biotechnology Information (NCBI) using the Basic Local Alignment Search Tool (Blast)^92^. Genus names of sequenced isolates were provided based on >99% DNA sequence similarity to reference strains in the database.

### Pairwise microbial interactions

Antagonistic interactions between bacteria and the *Escovopsis* were done in Petri dishes of 90 × 15 mm filled with 10 mL of mannitol soya flour medium (SFM)^89^. SFM was inoculated with 0.5 µL of the spore suspension of bacteria (~6 × 10^6^ CFU) and cultivated for ~3 days, spore of *Escovopsis* (~6 × 10^6^ CFU) were them inoculated at 5 mm from the border of the bacterial colony and the plates were cultivated for ~3 days at room temperature (at 25 °C). Control plates with single cultures were grown in parallel under the same conditions.

### Matrix-assisted laser desorption ionization imaging mass spectrometry (MALDI-IMS)

To perform MALDI-IMS a small section of SFM agar (20 mm^2^) containing the cultured microorganisms were cut and transferred to a MALDI MSP 96 anchor plate^93^. The fungal spores and aerial mycelia were removed using a cotton swab dampened in acetonitrile^71^, followed by dry deposition of universal matrix (1:1 mixture of 2,5-dihydroxybenzoic acid and α-cyano-4-hydroxy-cinnamic acid) over the agar using a 53 µm molecular sieve, plates were dried at 37 °C for 10 hours, and photograph was taken before MALDI analysis. Samples were analyzed using a Bruker Autoflex MALDI-TOF mass spectrometer (Bruker Daltonics, Billerica, MA, USA) or a Bruker Microflex MALDI-TOF mass spectrometer (Bruker Daltonics, Billerica, MA, USA) in positive reflectron mode, with 500 µm–600 µm laser intervals in X and Y directions, and a mass range of 60–2500 Da. Data obtained were analyzed using FlexImaging 3.0 software (Bruker Daltonics, Billerica, MA, USA). Resulting images were processed using ImageJ 1.47 V software (Research Services Branch, National Institute of Mental Health, Bethesda, MD, USA).

### Tandem mass spectrometry (MS/MS) fractionation experiments

Extracts of microbial interactions were obtained by macerating 20 mm^2^ of SFM agar in 1000 µL of ethyl acetate or methanol for three hours and centrifuged at 4000 rpm. The supernatant was removed and analyzed using an ion trap-Fourier transform ion cyclotron resonance mass spectrometer (IT-FTICRMS) or using electrospray ionization quadrupole- time of flight mass spectrometer (ESI-Q-TOF-MS).

For IT-FTICRMS analyses extracts were diluted 1:10 in electrospray mixture (49.5% MeOH, 49.5% H_2_O, 1% formic acid) and directly infused into the mass spectrometer 6.42 T Thermo Finnigan LTQ-FT-ICR (Thermo-Electron Corporation, San Jose, CA) using a TriVersa NanoMate chip-based electrospray ionization source (Advion Biosystems). Back pressure of 0.35–0.5 psi and spray voltage of 1.35–1.45 kV was used for the sample infusion. MS instrument was tuned with the 15+ charge state of Cytochrome C *m/z* 816 (isolation width: 1 *m/z*; activation Q: 0.25; activation time: 30 ms) using Tune Plus software version 1.0. Extracts and standards were run using QualBrowser software version 1.4 SR1 (Thermo) and consisted of one 10 minute segment, in which a high resolution FT profile mode scan cycled with four MS/MS data-dependent scans in the ion trap. MS/MS fragments were acquired for the four most intense ions of the FT scan by collision induced dissociation (CID) (isolation width: 2 *m/z*; activation Q: 0.25; activation time: 30 ms; Normalized collision energy: 35.0). After fragmentation the most intense ions were placed on a dynamic exclusion list for 600 s.

For ESI-Q-TOF-MS analyses extracts were diluted in methanol at 0.5 mg/mL and analyzed with an Agilent 1290 Infinity LC system (Agilent technologies, Santa Clara, CA) using a Kinetex® 1.7 µm C18 100 Å, LC Column 50 × 2.1 mm (Phenomenex Inc, Torrance, CA) and micrOTOF-Q III™ mass spectrometer (Bruker Daltonics, Billerica, MA, USA) supplied with a electrospray ionization source (ESI). The gradient for chromatographic separation was carried out using a step UPLC run of methanol and acidified water (99.9% water and 0.1% formic acid), 8 min gradient from 10% methanol and 90% acidified water to 100% methanol, held at 100% methanol for 8 min at a flow rate of 0.3 mL/min throughout the run. MS spectra were acquired in positive ion mode in the range of 50–2225 *m/z* using data depend MS/MS fragmentation as described by Garg *et al*.^94^. Prior to data collection an external calibration with Agilent ESI-L Low Concentration Tuning Mix (Agilent Technologies, Santa Clara, CA) was performed and throughout the runs we use reserpine (Sigma-Aldrich Co. LLC.) for lock mass calibration.

### Molecular networking

MS raw data obtained in the MS^2^ fractionation experiments were converted to 32 bit mzXML file using Bruker Compass Data analysis v4.1 (Bruker Daltonics, Billerica, MA, USA) or MSConvert GUI (ProteoWizard)^95^. Once all raw data from extracts and standards were converted to mzXML format, these were analyzed using mass spectral molecular networking^96, 97^, as described by Watrous *et al*. and Yang *et al*.^36, 39^. Data analysis included the following parameters: cosine threshold set at 0.6 value, precursor mass tolerance of 1.0 Da, fragment mass tolerance of 0.3 Da, minimum number of matched peaks per spectral alignment of 6, consensus spectra for cosine score higher than 0.95, parent mass tolerance of 1.0 Da, minimum percentage of overlapping masses between two spectra of 45%, minimum percentage of matched peaks in a spectral alignment of 40%, using global natural products social molecular networking platform (GNPS, http://gnps.ucsd.edu)^38^.

Visualization of the network as nodes and edges was done using Cytoscape 2.8.0^98^ by importing clustering data and MATLAB attributes of the network^98^. A graph-drawing force-directed algorithm that uses the Fast Multipole Multilevel Method (FM3) was applied to the data set to organize and align the nodes within the network^99^. Other visual parameters were adjusted using VizMappers™, the node colors were illustrated according to the source of the precursor ions, and the edge thickness attribute is an interpretation cosine similarity score, with thicker lines indicating higher similarity. Sub-networks were created from selected parts of the complete network to show details of node connectivity.

### Identification of metabolites

The purification of the compounds was carried out using solid phase extraction cartridge Supelclean™ LC-18 SPE Tube (Supelco Inc, Bellefonte, PA) eluted with a step gradient of 20%, 40%, 60%, 80%, and 100% of methanol in water, followed by HPLC separation using reverse phase silica gel column Synergi™ Fusion-RP (Phenomenex Inc, Torrance, CA), eluted with methanol:water, 40 min gradient from 60% methanol and 40% water to 100% methanol, held at 100% methanol for 20 min at a flow rate of 1 mL/min throughout the run.

NMR spectra were acquired on a Jeol Eclipse 400 MHz spectrometer and referenced to residual solvent ^1^H and ^13^C signals δ_H_ 3.31, δ_C_ 49.00 for Methanol-d6. Metabolites were dereplicated by comparing high resolution MS data using AntiMarin database (University of Canterbury, Christchurch, New Zealand) and chemical literature^23, 24, 26, 27^. Additionally, we annotated selected MS^2^ fractionation patterns of bacterial metabolites and comparing them with MS^2^ of commercial standards using GNPS public spectral libraries Version 1.2.8-GNPS (University of California at San Diego, La Jolla, CA)^38^.

### *In vitro* bioassays

Compounds isolated were tested in bioassays against the human breast cancer cell line MCF-7^100^, the malaria parasite (*Plasmodium falciparum*)^101, 102^ and the Chagas’ disease causative agent (*Trypanosoma cruzi*)^100, 103, 104^, *Escovopsis* ACRO424 and TZ49, *Aspergillus fumigatus* (ATCC® 1028™)^105^, *Candida albicans* (ATCC® 10231™), *Staphylococcus aureus* subsp. *aureus* (ATCC® 43300™) and *Bacillus subtilis* subsp. *subtilis* (ATCC® 6051™)^106, 107^ (See Supplementary methods).

### Data availability statement

All data generated or analyzed during this study are included in this article (and its Supplementary Information files). ﻿﻿MS data is deposited in the GNPS MassIVE repository under the accession number: MSV000081208.﻿

## Electronic supplementary material

Supplementary Information
