# Supplementary material for: Imaging mass spectrometry and MS/MS molecular networking reveals chemical interactions among cuticular bacteria and pathogenic fungi associated with fungus-growing ants
Source: Sci Rep. 2017 Jul 17;7:5604. doi: 10.1038/s41598-017-05515-6 (PMC5514151; doi:10.1038/s41598-017-05515-6)
Supplement: Supplementary file 1 — Supplementary Information [file 41598_2017_5515_MOESM1_ESM.pdf]

Supplementary Information

Imaging mass spectrometry and MS/MS molecular networking reveals chemical interactions among cuticular bacteria and pathogenic fungi associated with fungus-growing ants

Cristopher A. Boya P.[1,2], Hermógenes Fernández-Marín[1], Luis C. Mejía[1], Carmenza Spadafora[3], Pieter C. Dorrestein[4,5], and Marcelino Gutiérrez Guevara[1,*]

[1]Centro de Biodiversidad y Descubrimiento de Drogas, Instituto de Investigaciones Científicas y Servicios de Alta Tecnología (INDICASAT-AIP), Panamá, Apartado 0843-01103, República de Panamá;

[2]Department of Biotechnology, Acharya Nagarjuna University, Guntur, Nagarjuna Nagar-522 510, India.

[3]Centro de Biología Celular y Molecular de Enfermedades, INDICASAT-AIP, Panamá, Apartado 0843-01103, República de Panamá

[4]Collaborative Mass Spectrometry Innovation Center, Skaggs School of Pharmacy and Pharmaceutical Sciences, University of California at San Diego, San Diego, California 92093, United States

[5]Department of Pharmacology, University of California at San Diego, San Diego, California 92093, United Stated

*mgutierrez@indicasat.org.pa

Contents

Supplementary Methods

- Breast cancer bioassay
- *P. falciparum* bioassay
- *T. cruzi* bioassay
- Antibiotic susceptibility test
- Antifungal bioassay

Supplementary Figures

- **Figure S1.** MALDI imaging mass spectrometry experiment of the interaction between the bacterium *Streptomyces* CBR53 and the fungus *Escovopsis* TZ49, showing ions from 50 *m/z* to 800 *m/z*
- **Figure S2.** MALDI imaging mass spectrometry experiment of the interaction between the bacterium *Streptomyces* CBR53 and the fungus *Escovopsis* TZ49, showing ions from 800 *m/z* to 2500 *m/z*
- **Figure S3.** Proton NMR spectra of elaiophylin macrolide, ^{1}H NMR (400 MHz, METHANOL-d_3)
- **Figure S4.** Carbon NMR spectra of elaiophylin macrolide, ^{13}C NMR (100 MHz, METHANOL-d_3)
- **Figure S5.** Direct MS-MS comparison of elaiophylin macrolide from the extract of *Streptomyces* sp. (CBR53) with elaiophylin-CDM bronze spectrum from GNPS spectral library (CCMSLIB00000479766)
- **Figure S6.** Efomycin G from the extract of Streptomyces CBR53.
- **Figure S7**. Efomycin A from the extract of Streptomyces CBR53.
- **Figure S8.** MS-MS comparison of unknown peaks 1065.6007, 1079.6139 and 1093.6222 from the extract of *Streptomyces* CBR53, with elaiophylin-CDM bronze spectrum from GNPS spectral library (CCMSLIB00000479766).
- **Figure S9.** Direct MS-MS comparison of shearinine D from the extract of *Escovopsis TZ49* with silver spectrum of Shearinine D_130028 from GNPS spectral library (CCMSLIB00000478461)
- **Figure S10**. Direct MS-MS comparison of shearinine F from the extract of *Escovopsis* TZ49 with silver spectrum of Shearinine F_120146 from GNPS spectral library (CCMSLIB00000478066)
- **Figure S11:** MALDI imaging mass spectrometry experiment of the interaction between the bacterium *Streptomyces* CBR38 and the fungus *Escovopsis* Acro424, showing ions from 50 *m/z* to 2500 *m/z*
- **Figure S12:** Direct MS-MS comparison of actinomycin D from the extract of *Streptomyces* CBR38 with actinomycin D gold spectrum from GNPS spectral library (CCMSLIB00000006871)
- **Figure S13:** Direct MS-MS comparison of actinomycin X2 from the extract of *Streptomyces* CBR38 with actinomycin X2 gold spectrum from GNPS spectral library (CCMSLIB00000577607)

- **Figure S14**. Direct MS-MS comparison of ion 1271.6415 from the extract of *Streptomyces* CBR38

Supplementary Tables

- ***Table S1.*** ^{13}C and ^{1}H NMR Chemical shifts of compound 1 (400 MHz, DMSO-d6, δ in ppm relat*ive to solvent) and elaiophylin data from literature*[1] (360 MHz, DMSO-d6, δ in ppm relative to internal TMS)

Supplementary Methods

Breast cancer bioassay

Activity against the human breast cancer cell line MCF-7 (ATCC, Manassas, VA, USA) was done as described by Higginbotham et al.[2]. In short, the cells were incubated with RPMI-1640 supplemented with gentamicin, L-glutamine, $NaHCO_3$, HEPES buffer, and FBS at 37°C. Compound was diluted in RPMI-1640 media, added to the cells, and incubated for 48 h at 37°C. After the cells were fixed with trichloroacetic acid, rinsed with water and treated with sulphorhodamine B, the mixture was allowed to react for 15–30 min at 22°C. Then the cells were rinsed with trichloroacetic acid, dried, and treated with Tris-HCl (10 mM; pH 7) for 15 min. Finally color intensity was read at 570 nm, in a color plate reader (Benchmark Bio-Rad). Adriamycin diluted in DMSO was used as positive control (normal IC_{50} value 20–50 nM).

Plasmodium falciparum bioassay

Activity against the causative agent of malaria was performed by culturing human erythrocytes and infecting them with *P. falciparum*, as described by Trager and Jensen, 1976[3]. Briefly, the HB3 (Chloroquine sensitive) strain of *P. falciparum* was cultured in RPMI 1640, supplemented with 10% human serum (from O+ blood) at a hematocrit of 2% human erythrocytes (O+) at 37°C in a gas mixture of 5% CO_2, 5% O_2, and 90% N_2. Parasites were kept in the same phase of the life cycle through synchronization in a temperature cycling incubator[4]. The parasites were initially tested with 10 µg/mL of the extract containing the compound, while the IC_{50} was obtained by adding no more than 10% v/v of the compound to the well, at different concentrations. The culture was incubated further for 24 h after which PicoGreen DNA fluorescent dye (Invitrogen, USA) was added to a final concentration of 1%. After 30 min incubation the signal was read on a fluorescence plate reader[5]. Chloroquine was used as reference positive control (average IC_{50}: 24.3 nM).

Trypanosoma cruzi bioassay

Activity against the causative agent of Chagas disease was performed using a colorimetric method, assessing Tulahuen LacZ clone C4 of *T. cruzi* parasites expressing β galactosidase (ATCC, Manassas, VA, USA)[6]. Concisely, assays were performed in duplicate on amastigotes culture in RPMI-1640 supplemented with L-glutamine, HEPES buffer, $NaHCO_3$, dilution of a penicillin-streptomycin mix (1:100) and FBS at 37°C, them exposed to different concentrations of the test compounds under an atmosphere of 5% CO_2/95% air[2]. Cleavage of chlorophenol red-β-D-galactoside (CPRG, Roche Applied Science) by β-Gal expressed by the parasite was measured at 570 nm to

detect color intensity, in a color plate reader (Benchmark Bio-Rad). Nifurtimox diluted in RPMI-1640 medium was used as a positive control (IC_{50} 0.15–13.4 µM)[2,6,7].

Antibiotic susceptibility test

Assays against strains of *Candida albicans* (ATCC® 10231™), *Staphylococcus aureus* subsp. *aureus* (ATCC® 43300™) and *Bacillus subtilis* subsp. *subtilis* (ATCC® 6051™) were performed using disk diffusion test, in duplicate as described by Boned[8] with some modifications[9]. Briefly, petri dishes with Müeller-Hinton agar were inoculated with a suspension of each test organism equivalent to 0.5 McFarland solutions, followed by the application of 6 mm papers disk on the surface of the agar and then impregnated with 25 µL of the compound at a concentration of 1024, 768, 512, 384, 256 and 128 µg/mL. Petri dishes were incubated at 30ºC for 18-24 hours before inhibitions zone were measured. MIC values were determined using regression analysis of inhibition zones, considering the absorptive model of diffusion[8].

Antifungal bioassay

Assays against fungal strains *Escovopsis sp*. (CBR53 and CBR38), *A. fumigatus* (ATCC® 1028™) were performed in duplicate using 6 mm papers disk containing three different concentrations of the compounds (1024, 512, and 256 µg/Ml). Hyphae plugs of fungal strains were placed in the center of the petri dishes containing Müeller-Hinton agar and incubated at 30ºC, evaluation of inhibition zones were carried out at 24, 48, 72, 96 and 120 hours[10].

Supplementary Figures

Figure S1. MALDI imaging mass spectrometry experiment of the interaction between the bacterium *Streptomyces* CBR53 and the fungus *Escovopsis* TZ49, showing ions from 50 m/z to 800 m/z

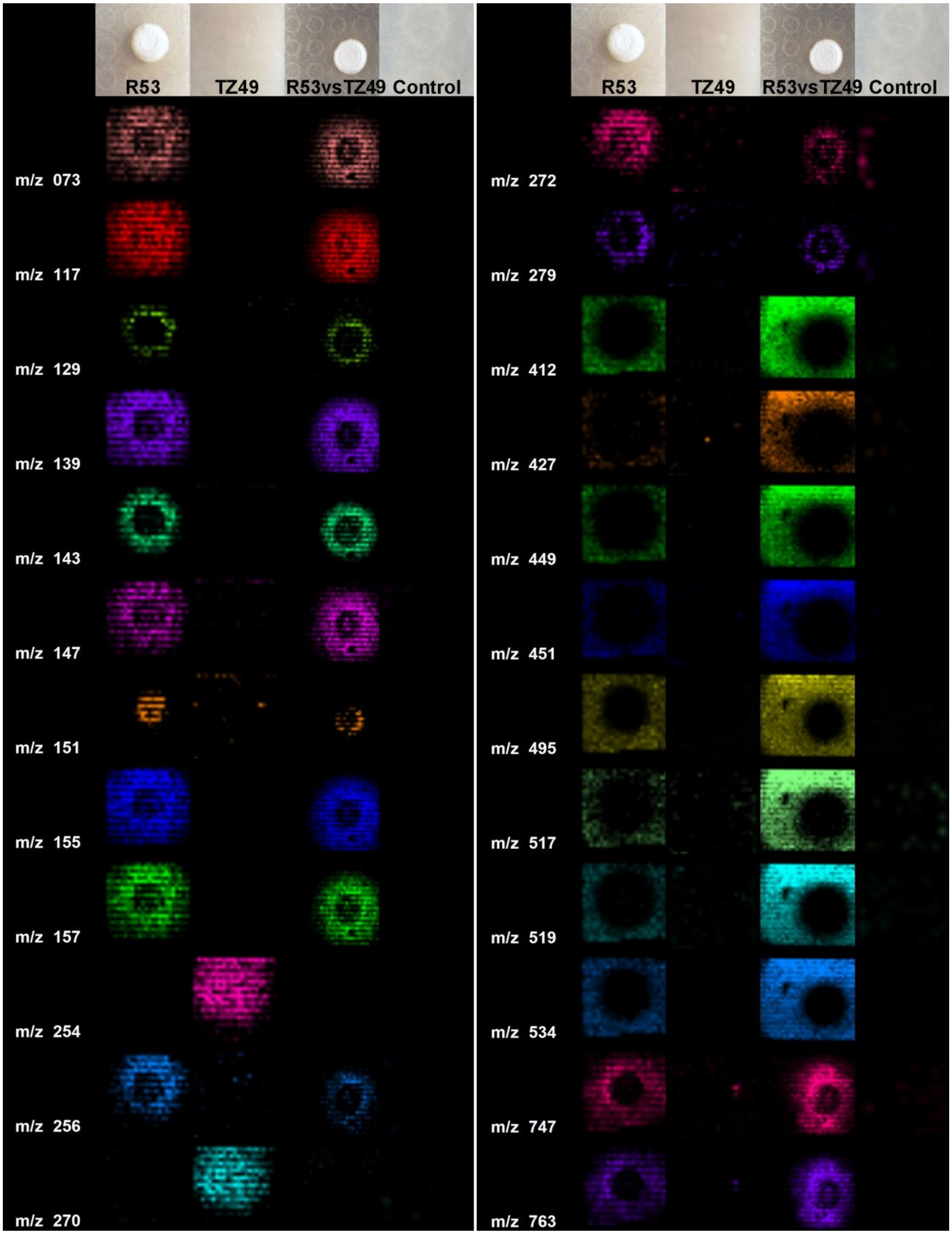

Figure S2. MALDI imaging mass spectrometry experiment of the interaction between the bacterium *Streptomyces* CBR53 and the fungus *Escovopsis* TZ49, showing ions from 800 m/z to 2500 m/z

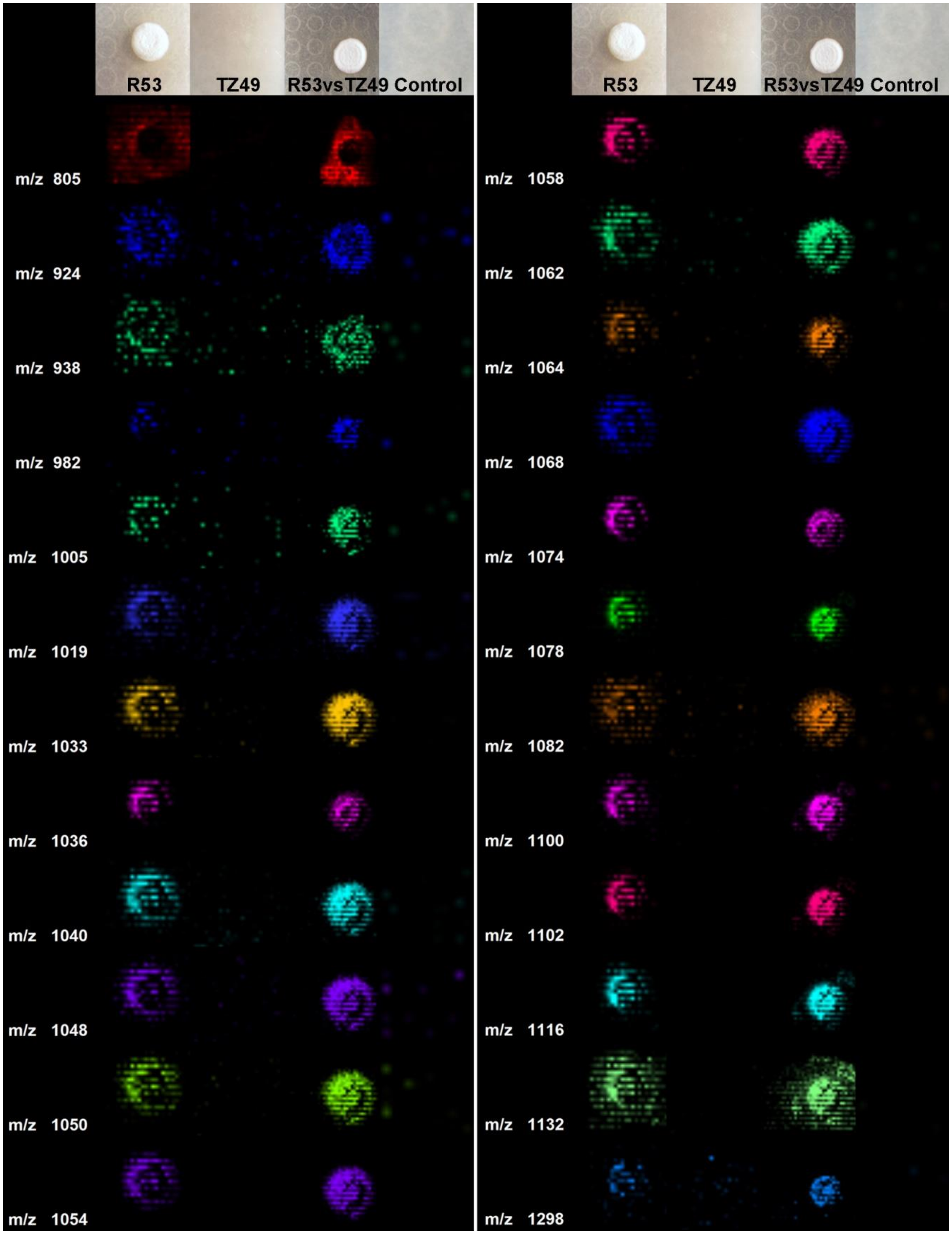

Figure S3. ^{1}H-NMR spectra of elaiophylin (400 MHz, DMSO-d6)

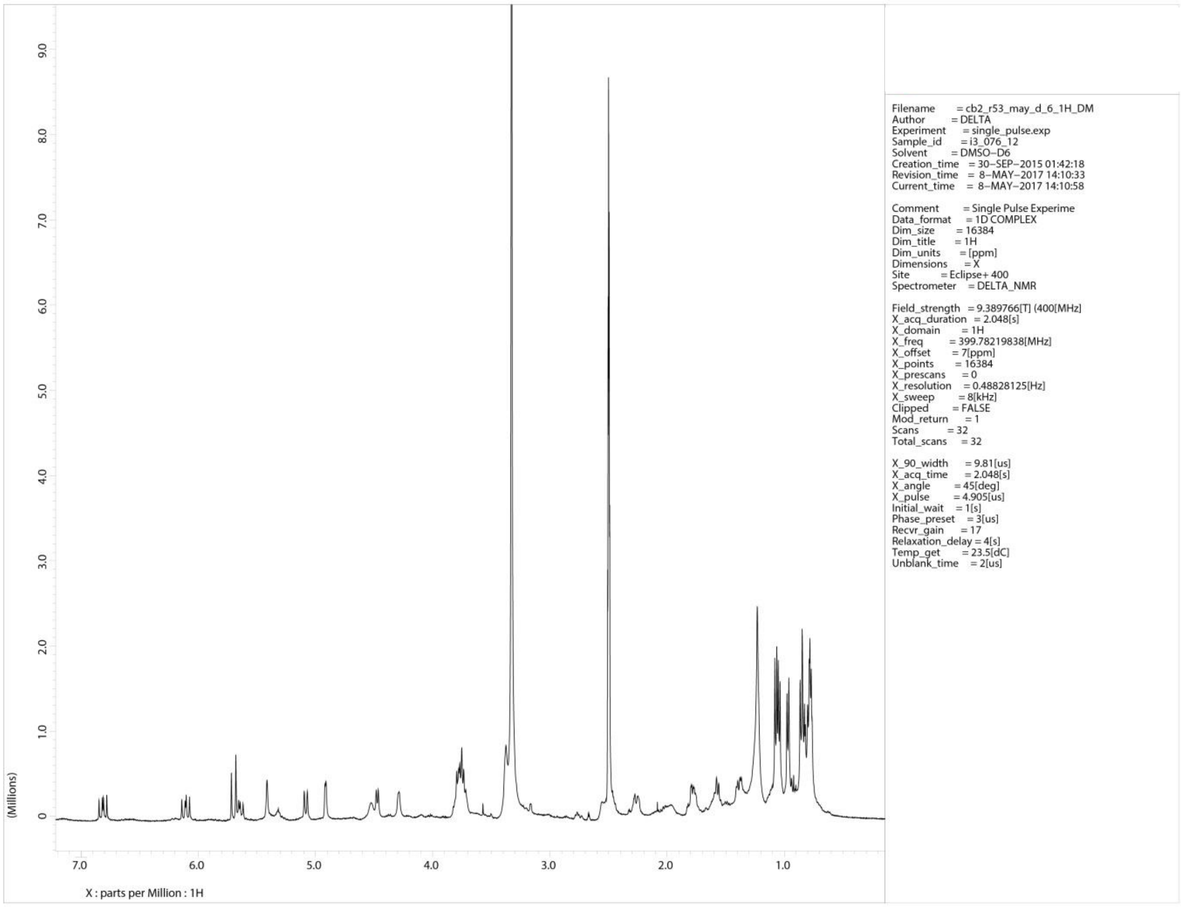

Figure S4. ^{13}C-NMR spectra of elaiophylin (100 MHz, DMSO-d6)

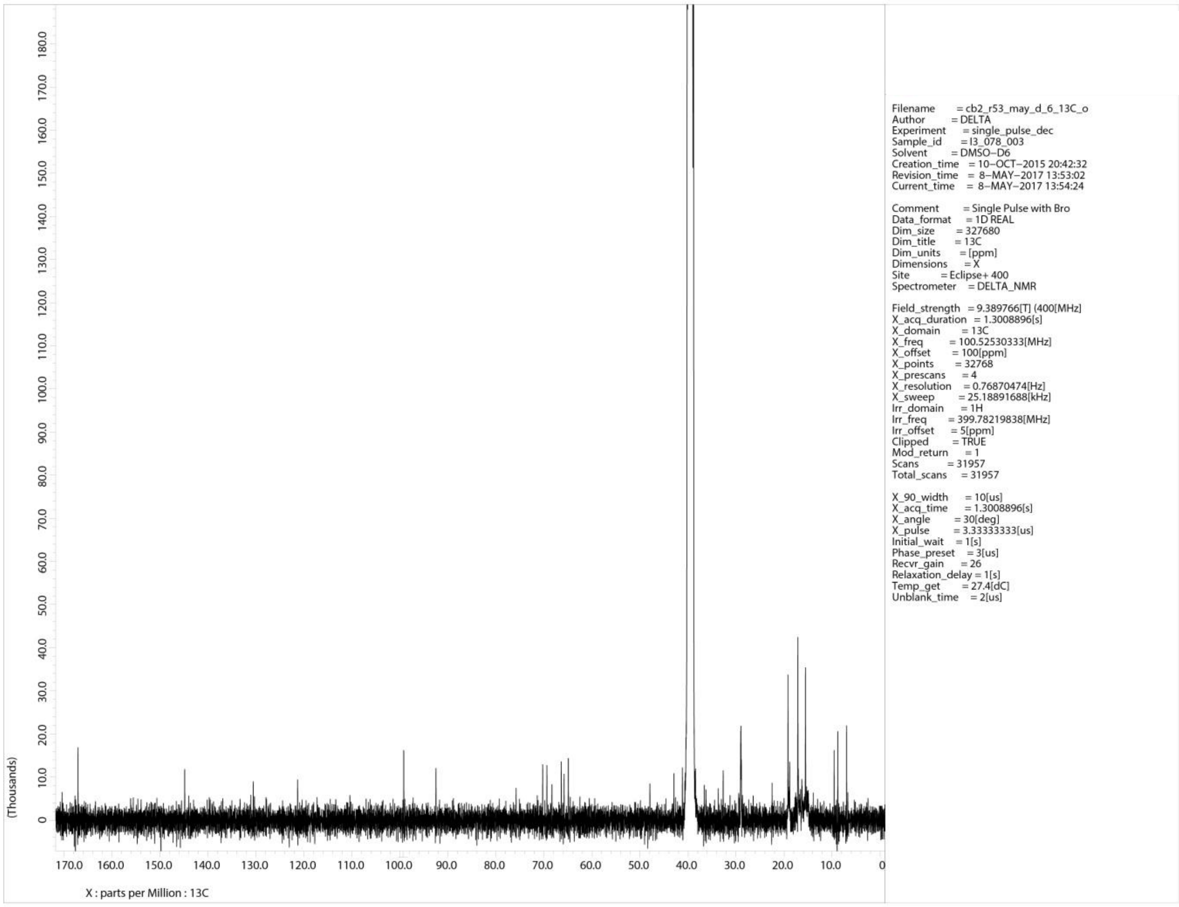

Figure S5. Direct MS-MS comparison of elaiophylin macrolide from the extract of *Streptomyces* CBR53 with elaiophylin-CDM (bronze spectrum) from GNPS spectral library (CCMSLIB00000479766). Picture show a singlet fragment at 729.3878 Da, which correspond to the fragmentation of the symmetrical groups bonded to the macrolactone ring of elaiophylin.

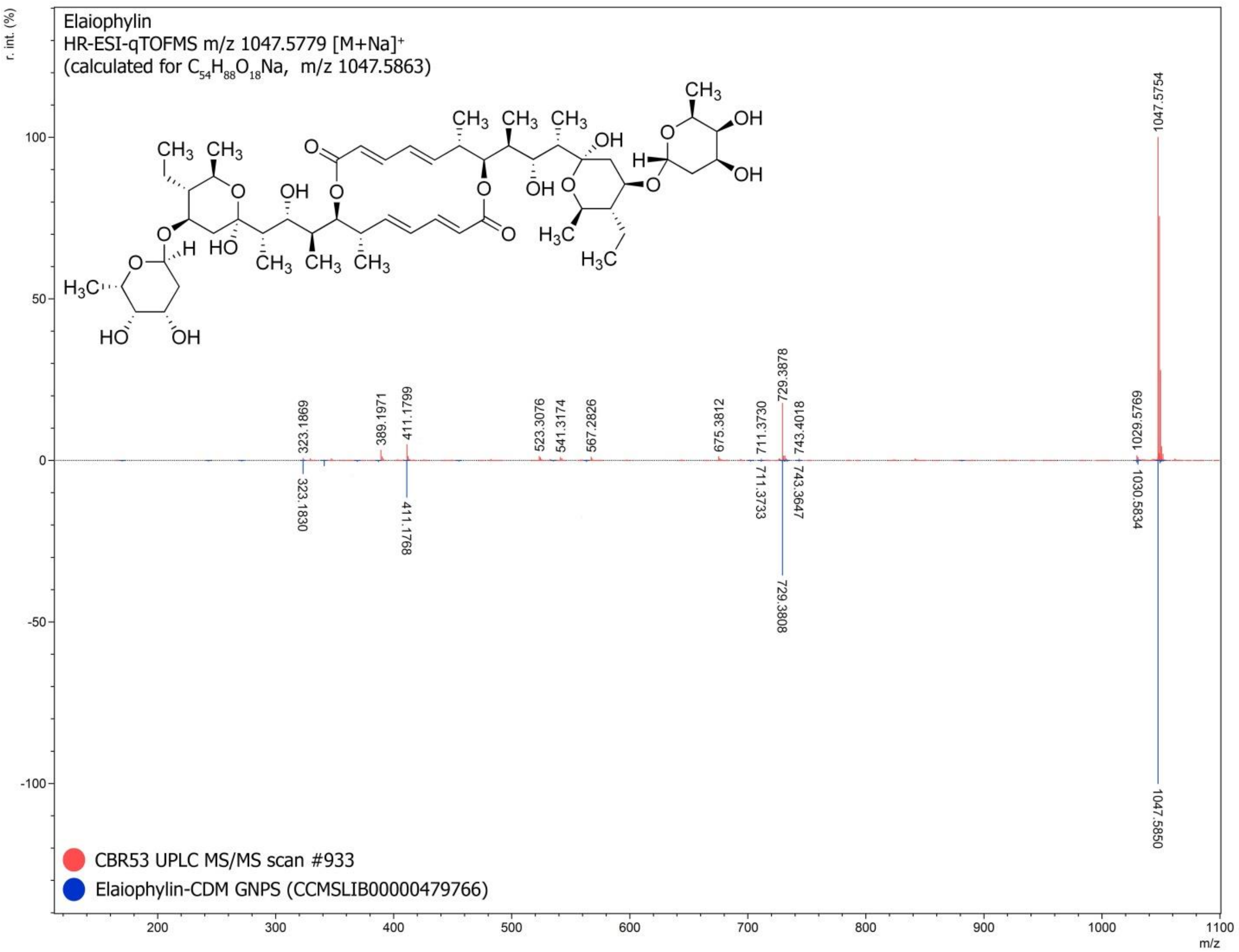

Figure S*6*. Efomycin G from the extract of *Streptomyces* CBR53. The spectrum show three mayor fragment ions: 411.1819 m/z which correspond to the aglycone; and the ions 715.3734, 729.3860 m/z, which indicate a cleavage of the nonsymmetrical groups between carbons 9-10. and 9''-10''.

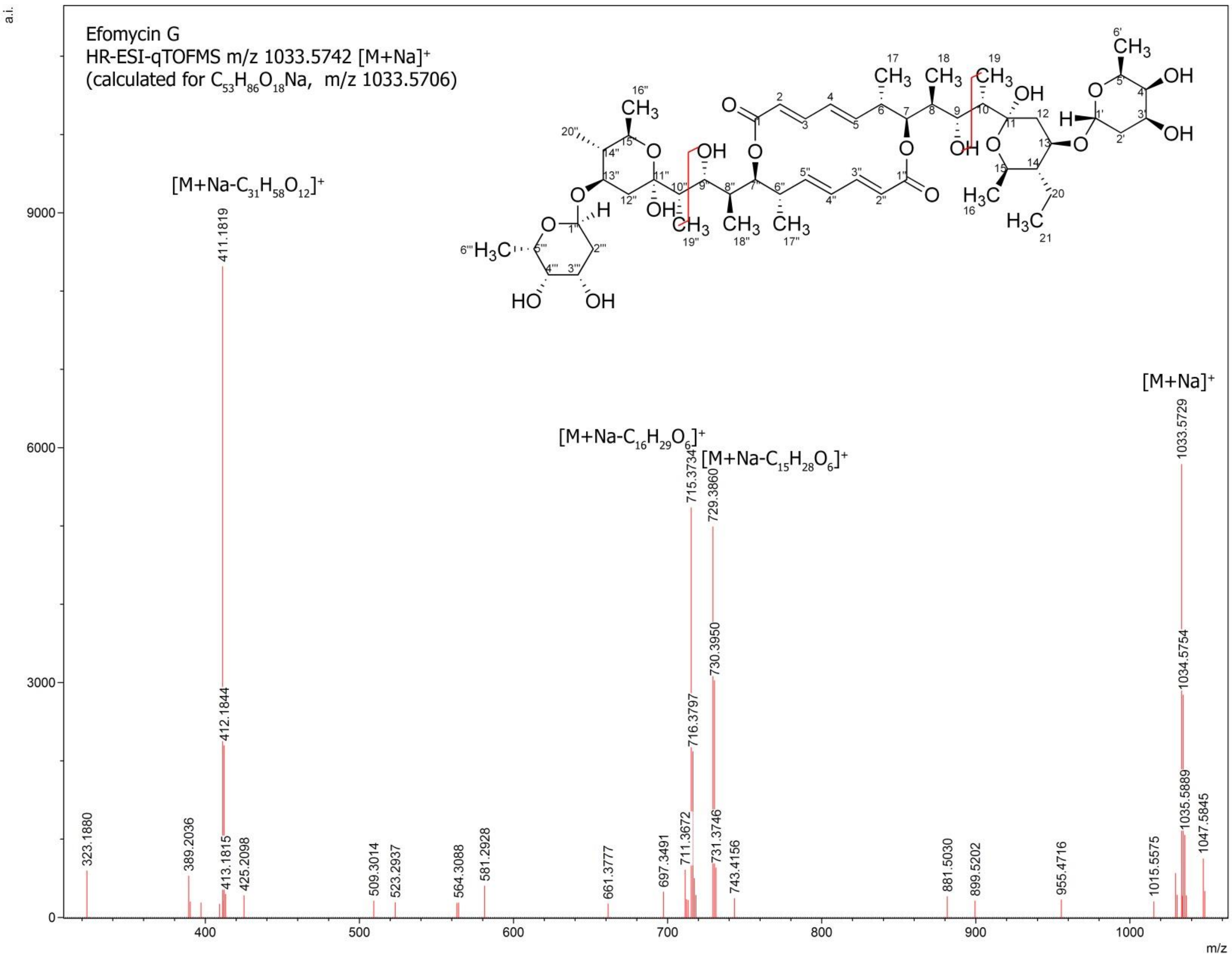

Figure S7. Efomycin A from the extract of *Streptomyces* CBR53. The spectrum show three mayor fragment ions: 411.1800 m/z which correspond to the aglycone; and the ions 743.3699, 729.3763 m/z, which indicate a cleavage of the nonsymmetrical groups between carbon 9-10 and 9’’-10’’.

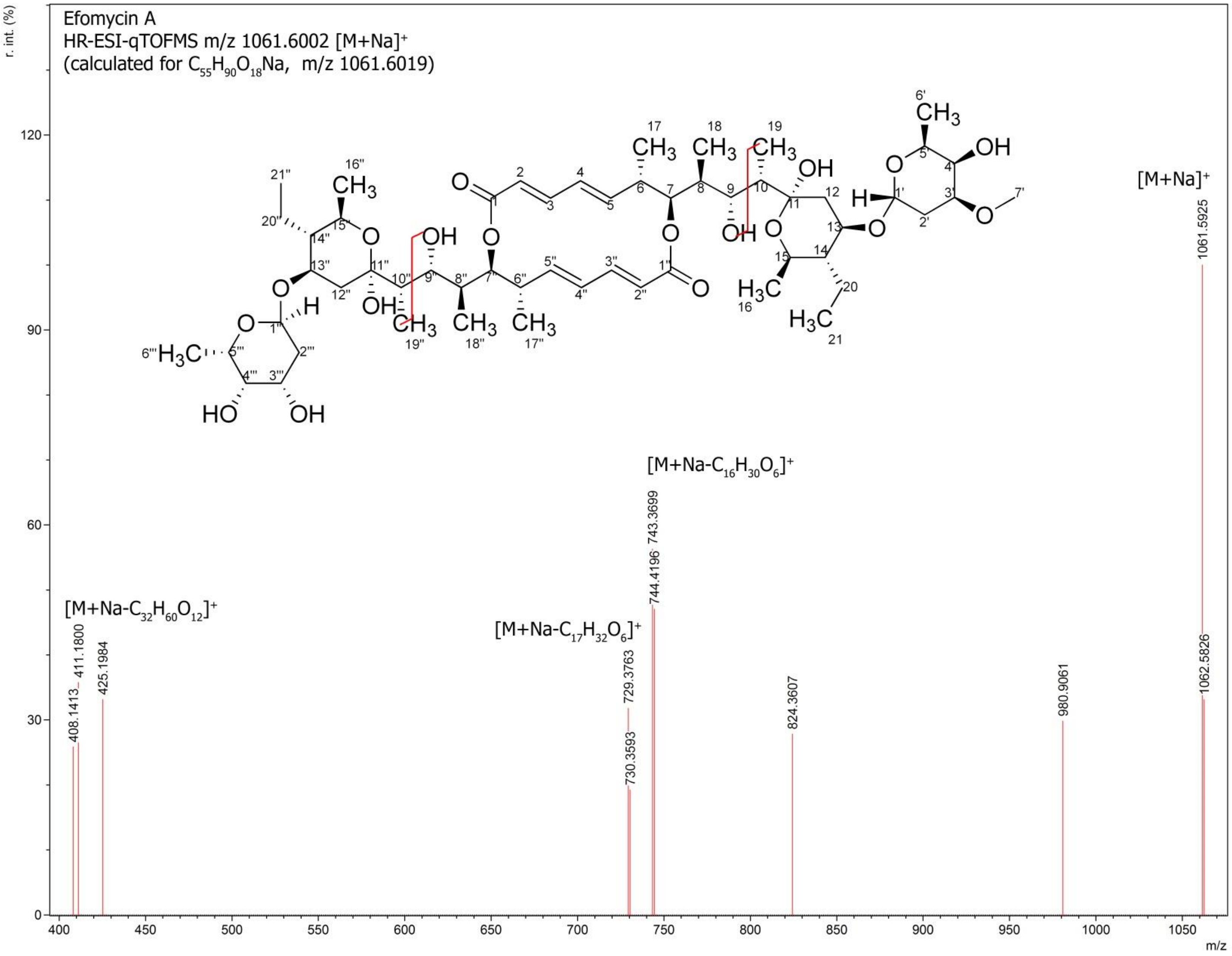

Figure S8. MS-MS comparison of unknown nodes 1065.6007, 1079.6139 and 1093.6222 from the extract of *Streptomyces* CBR53, with elaiophylin-CDM bronze spectrum from GNPS spectral library (CCMSLIB00000479766). Spectra show chemical shifts of 18, 32 and 46 Dalton related to elaiophylin.

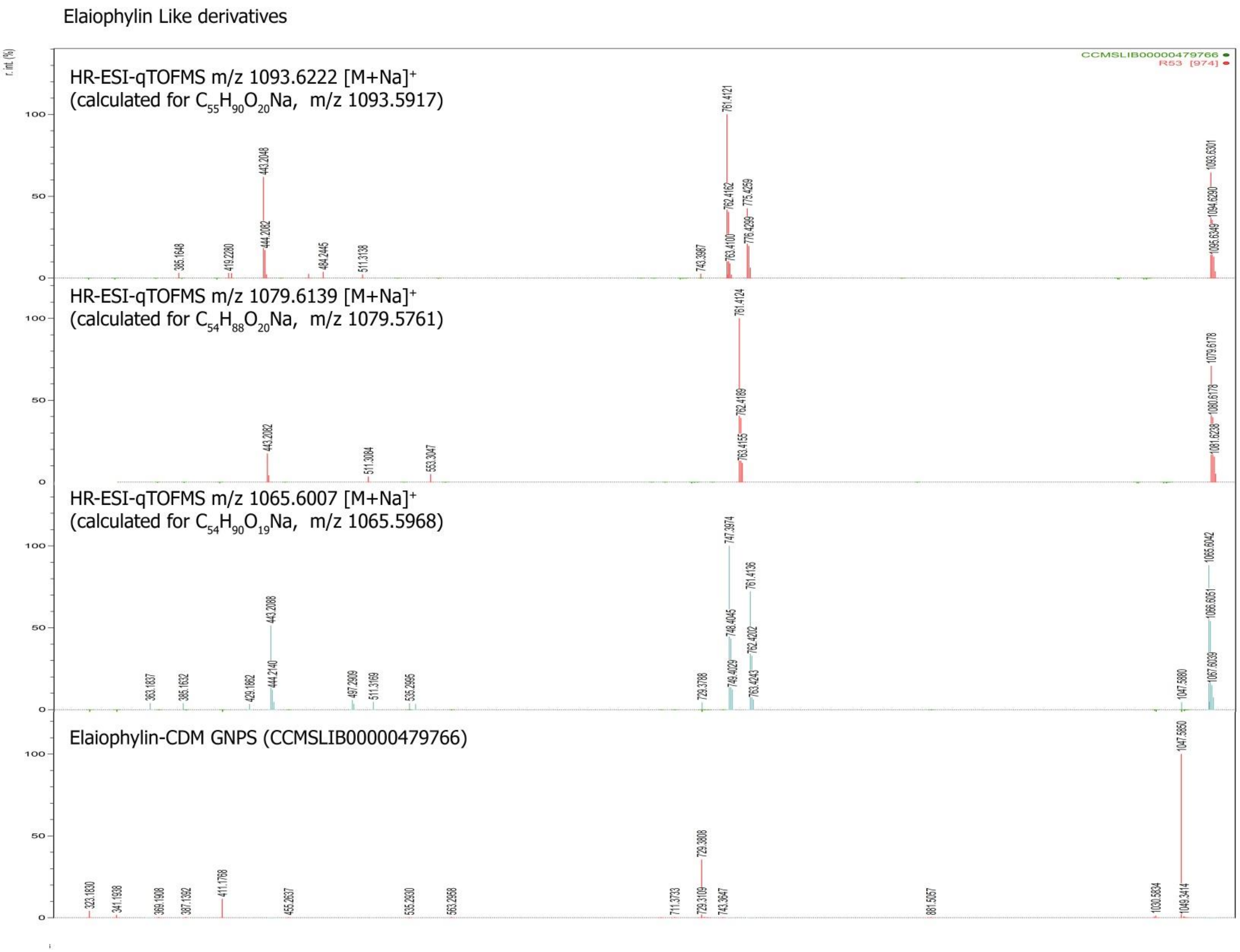

Figure S9. Direct MS-MS comparison of shearinine D from the extract of *Escovopsis* TZ49 with silver spectrum of Shearinine D_130028 from GNPS spectral library (CCMSLIB00000478461)

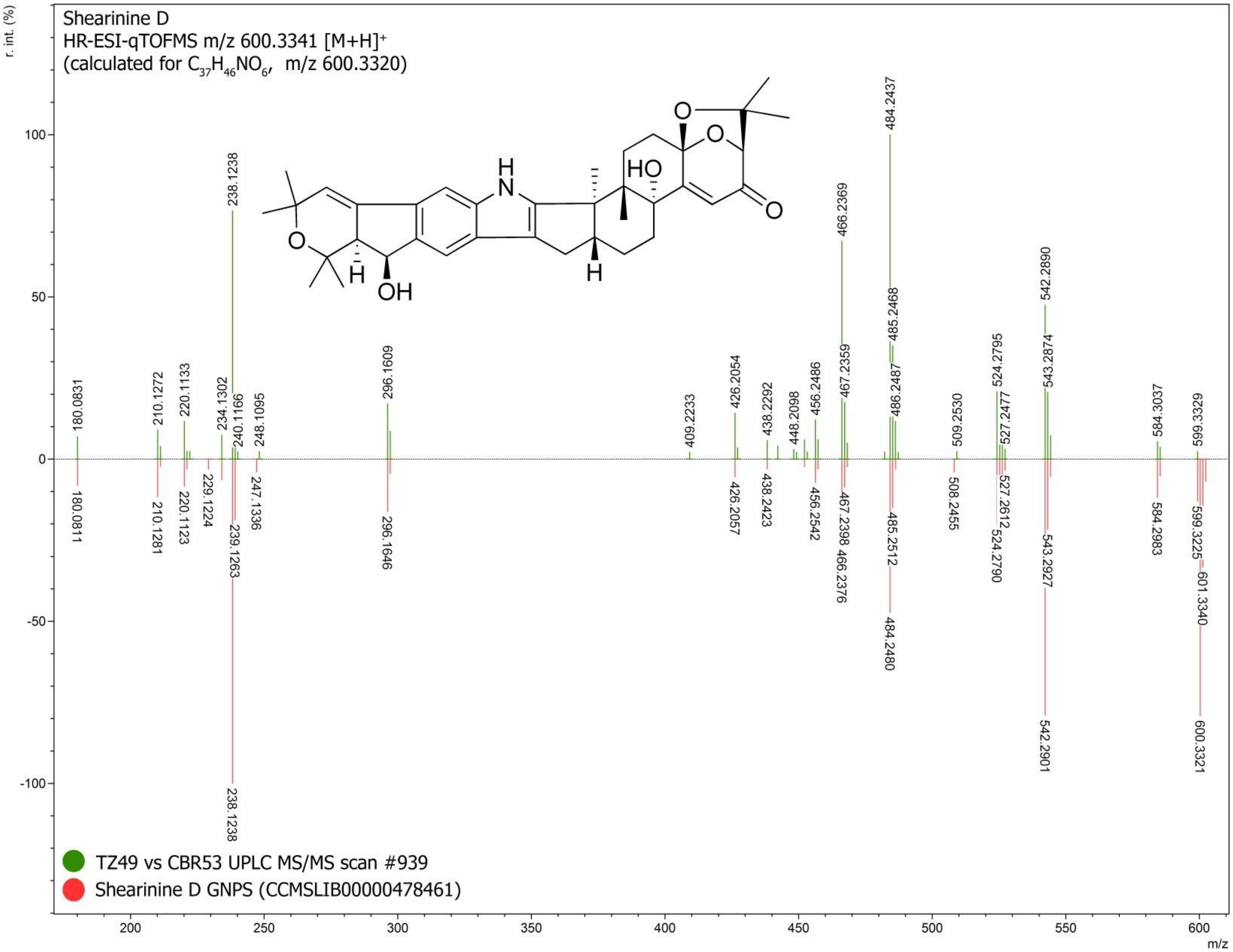

Figure S10. Direct MS-MS comparison of shearinine F from the extract of *Escovopsis* TZ49 with silver spectrum of Shearinine F_120146 from GNPS spectral library (CCMSLIB00000478066)

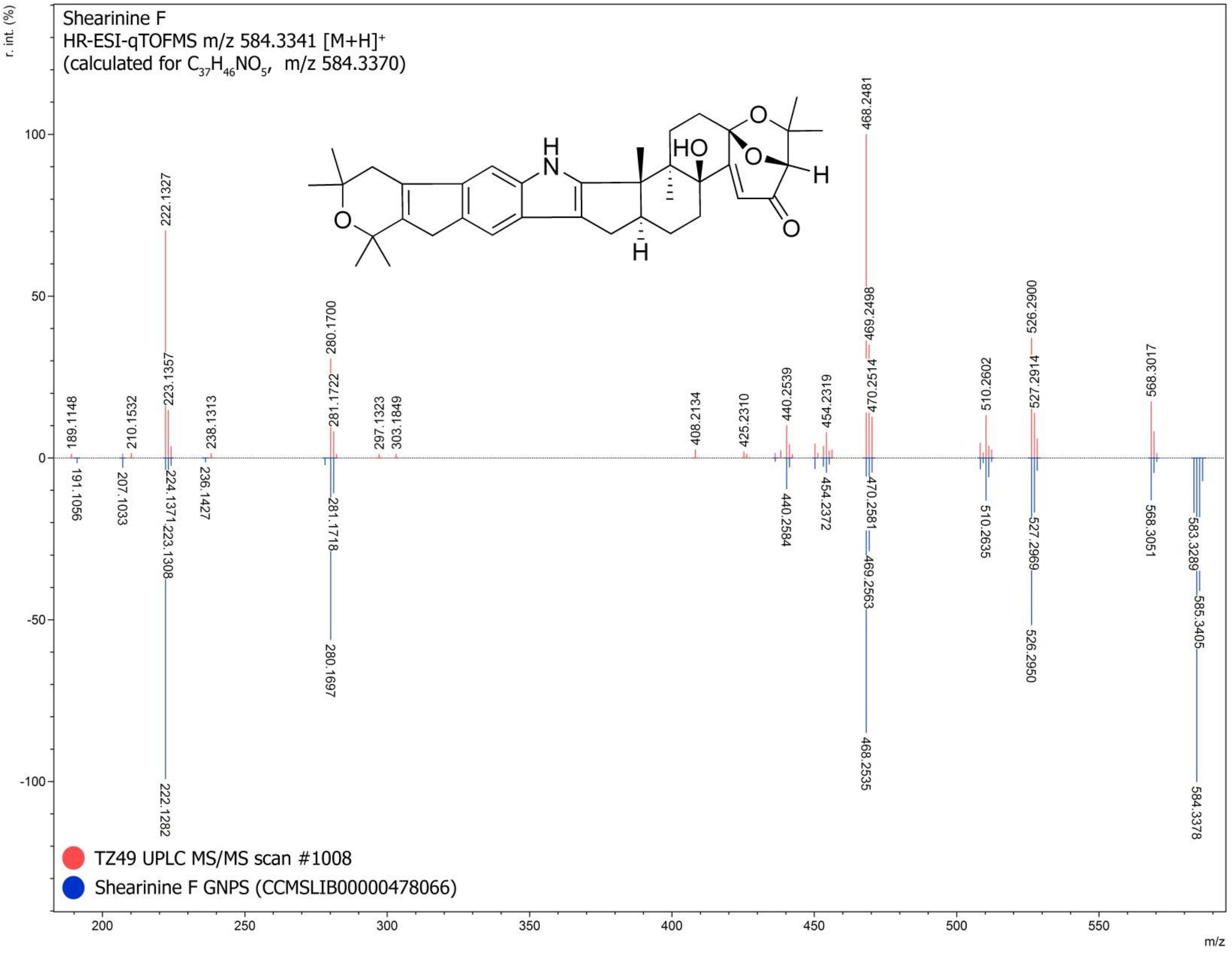

Figure S11. MALDI imaging mass spectrometry experiment of the interaction between the bacterium *Streptomyces* CBR38 and the fungus *Escovopsis* ACRO424, showing ions from 50 m/z to 2500 m/z

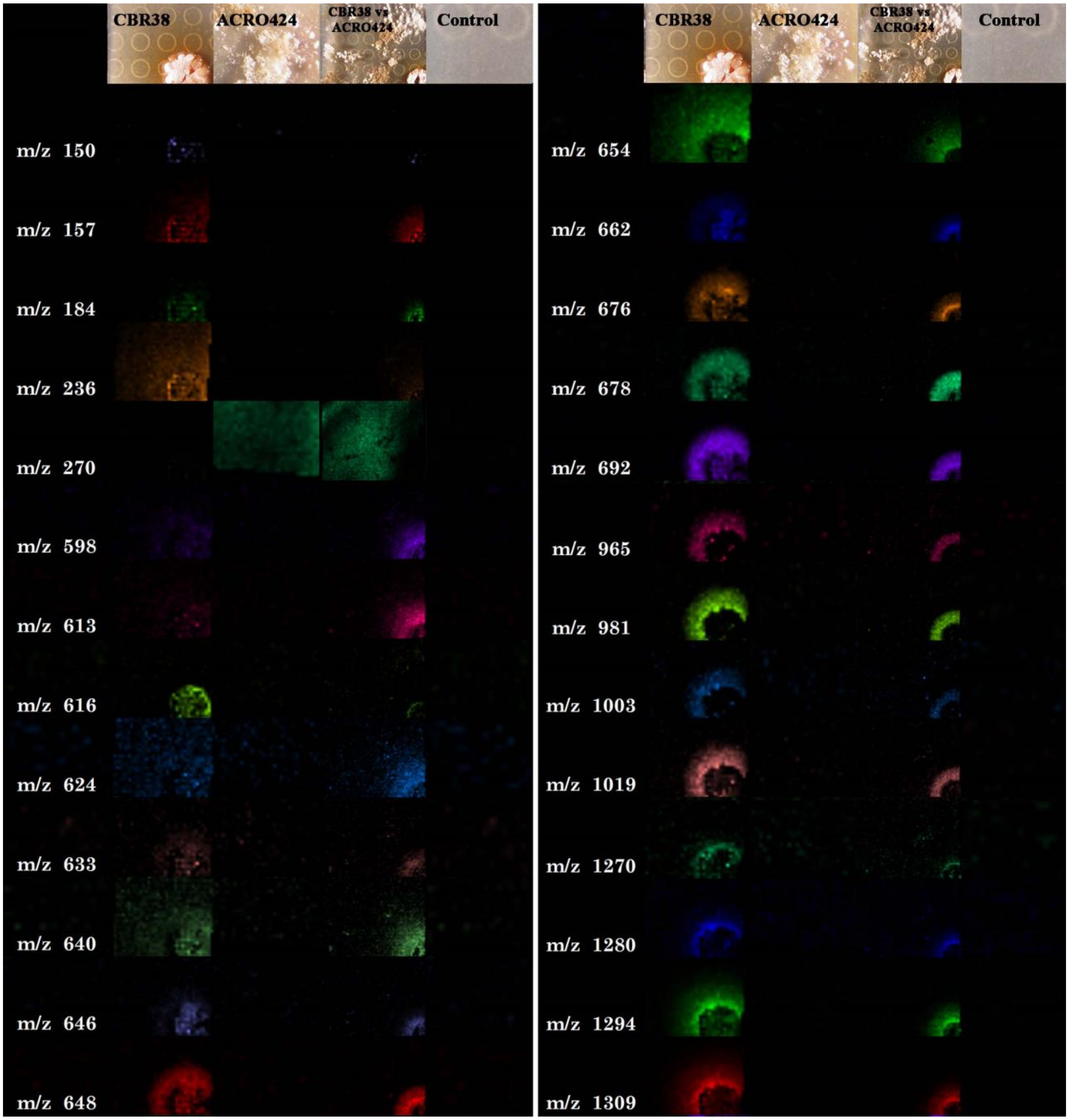

Figure S12. Direct MS-MS comparison of actinomycin D from the extract of *Streptomyces* CBR38 with actinomycin D gold spectrum from GNPS spectral library (CCMSLIB00000006871)

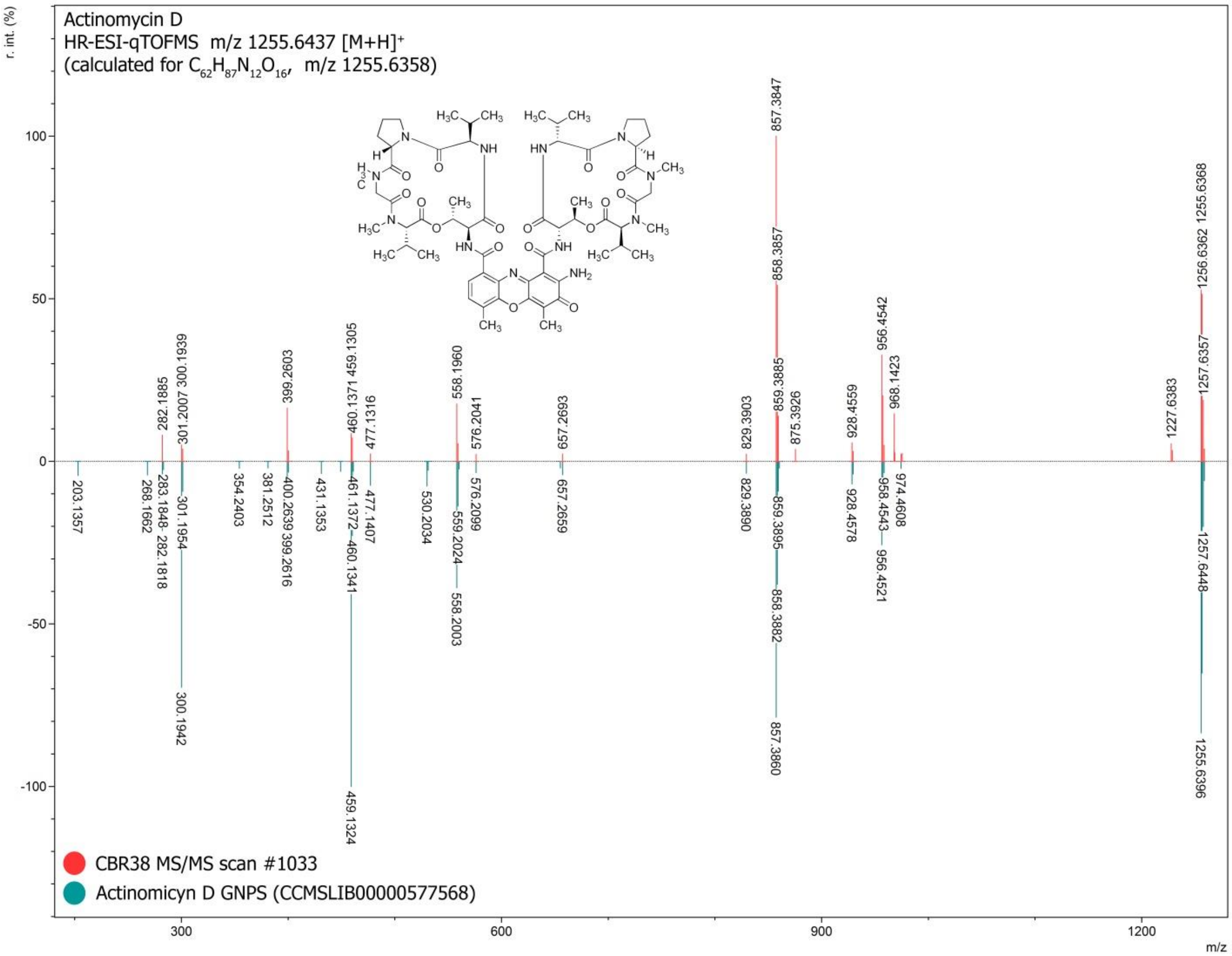

Figure S13. Direct MS-MS comparison of actinomycin X2 from the extract of *Streptomyces* CBR38 with actinomycin X2 gold spectrum from GNPS spectral library (CCMSLIB00000577607)

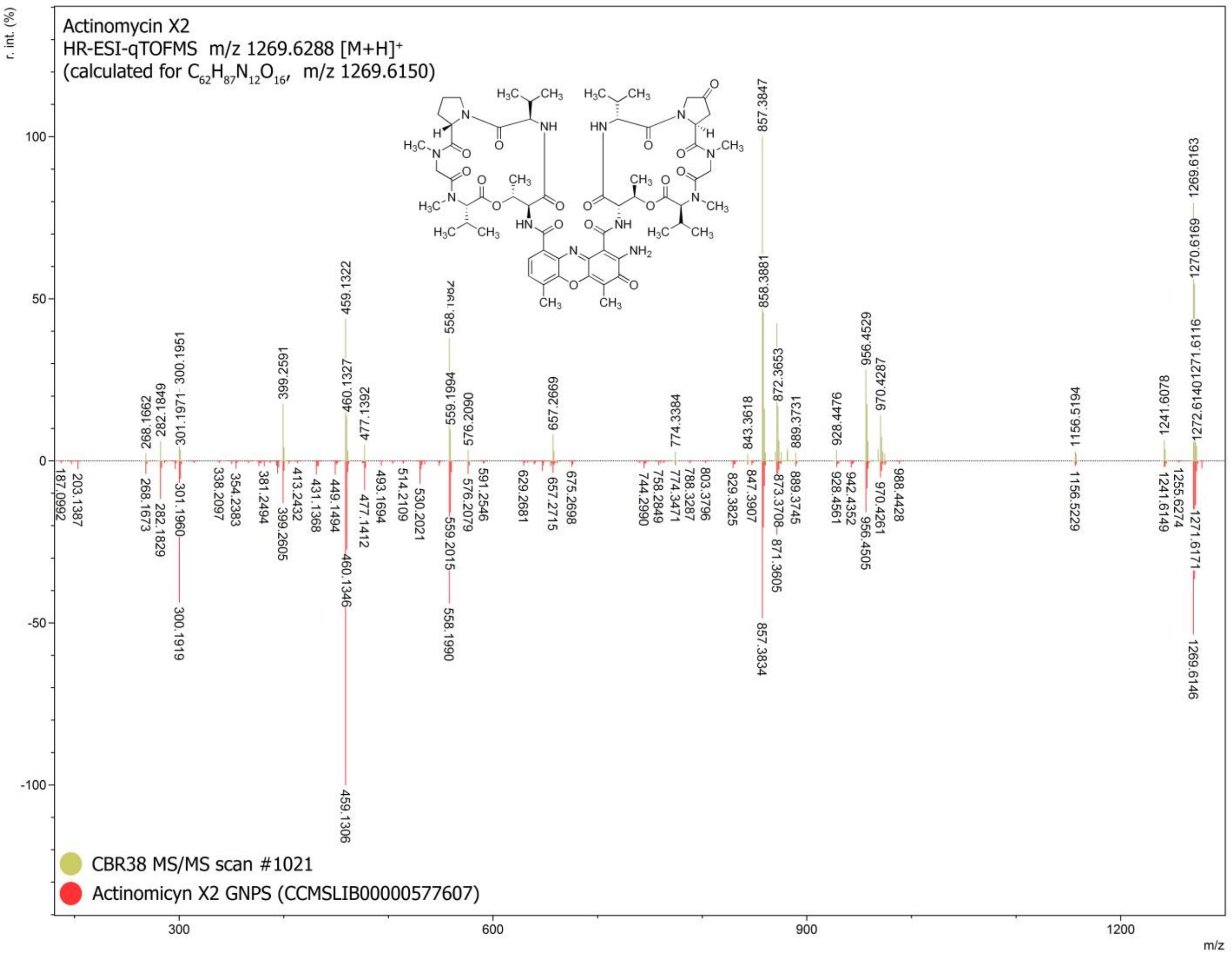

Figure S14. Direct MS-MS comparison of ion 1271.6415 with actinomycin $X_{0\beta}$ (CCMSLIB00000577768), result show twenty five shared peak (25) and less than 10 ppm of error for the calculated molecular formula.

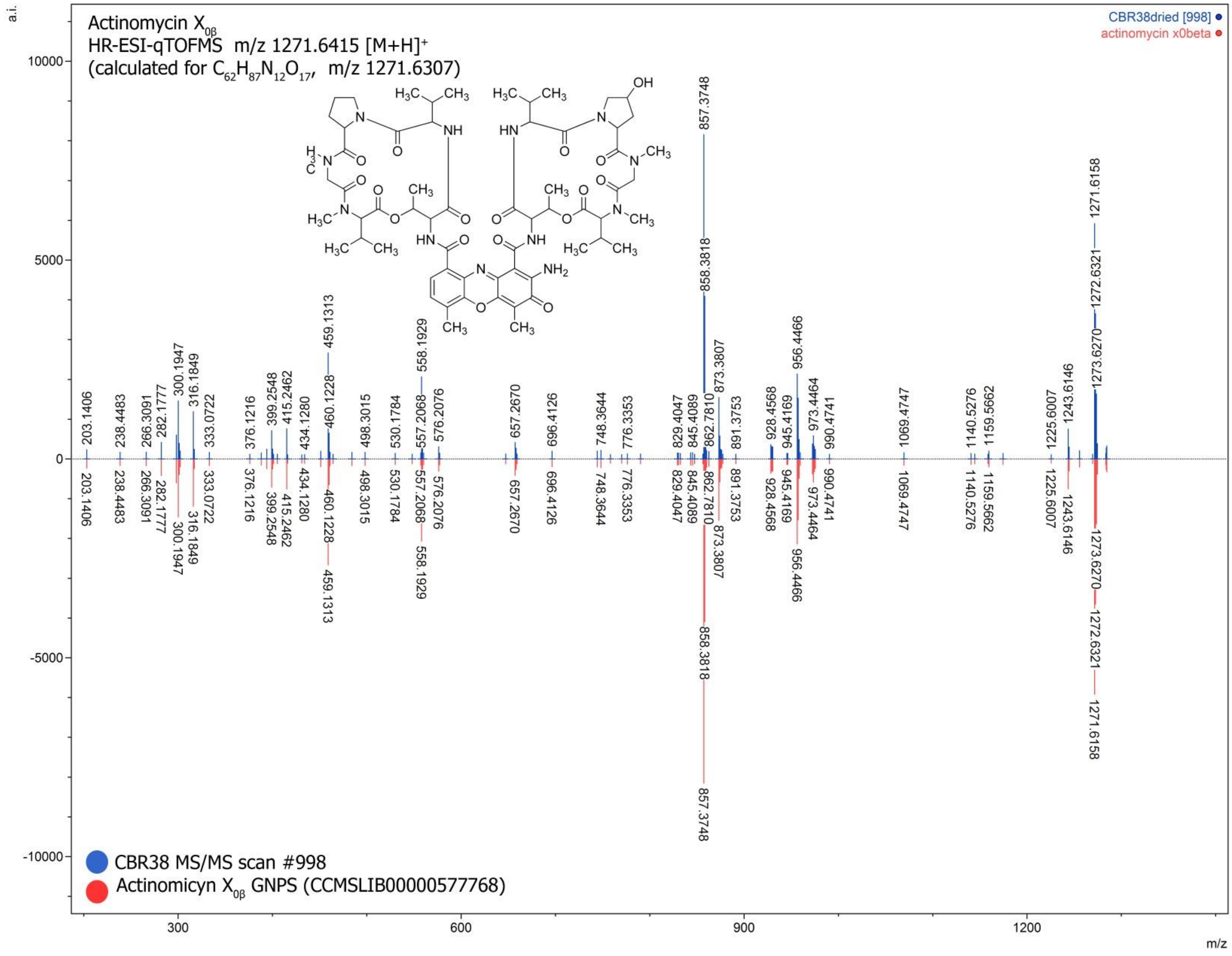

Supplementary Table

Table S1. Comparison of ^{13}C and ^{1}H NMR spectra of compound 1 (400 MHz, DMSO-d6, δ in ppm relative to solvent) and elaiophylin data from literature[1] (360 MHz, DMSO-d6, δ in ppm relative to internal TMS); Carbon numbers represent the lower and upper half portion of the molecule.

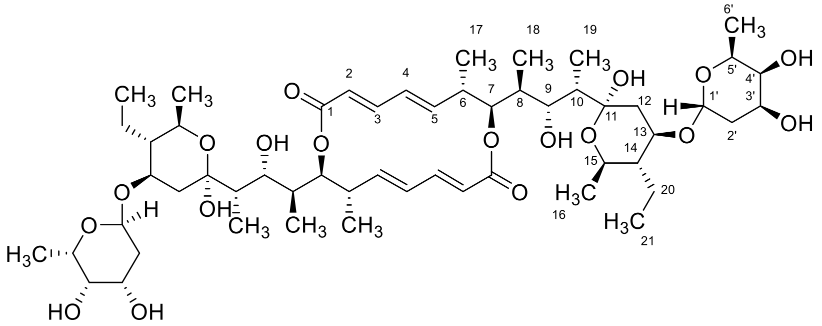

Carbon	Compound 1, δ_C	Elaiophylin, δ_C	Compound 1, δ_H (m, J in Hz)	Elaiophylin δ_H (m, J in Hz)
1	167.0966	167.22	-	--
2	121.3408	121.33	5.70 (d, *J*=15.14)	5.66 (d, *J*=15)
3	144.8549	144.86	6.82 (dd, *J*=15.38, 10.99)	6.80 (dd, *J*=15, 11)
4	130.5782	130.65	6.11 (dd, *J*=15.14, 11.23)	6.08 (dd, *J*=15, 11)
5	144.8549	144.86	5.64 (m)	5.61 (dd, *J*=16, 10)
6	41.1816	41.26	2.50 (m)	2.50 (m)
7	75.8113	75.9	5.09 (d, *J*=10.25)	5.06 (d, J=11)
8	36.2769	36.31	1.80 (m)	1.82 (m)
9	69.4062	69.53	3.76 (m)	3,70 (m)
10	42.9404	42.82	1.59 (m)	1.6 (m)
11	99.2291	99.19	--	--
12	36.6180	36.98	2.27 (m)	2.27 (m)
13	66.4224	66.42	3.76 (m)	3,70 (m)
14	47.9491	48.05	1.08 (d, *J*=6.35)	1.1 (m)
15	65.8198	65.93	3.76 (m)	3,70 (m)
16*	19.1800	19.14	1.05 (d, *J*=5.86)	1.07 (m)
17*	15.5370	15.49	0.97 (d, *J*=6.84)	1.01 (d, *J*=6)
18	8.7940	8.81	0.81 (m)	0.83 (d, *J*=6)
19	6.9848	6.90	0.81 (m)	0.83 (d, *J*=6)
20	19.1800	19.14	1.38 (d, *J*=4.88)<br>1.61 (m)	1.41 (m)<br>1.60 (m)
21	9.5663	9.47	0.81 (m)	0.83 (d, *J*=6)
1'	92.5197	92.68	4.92 (d, *J*=3.42)	4.90 (d, *J*=4Hz)
2'	32.6890	32.7	1.40 (m);<br>1.80 (m)	1.41(m)<br>1.82 (m)
3'	64.9420	65.04	3.76 (m)	3,70 (m)
4'	70.2872	70.39	3.38 (br. s.)	3.36 (br.s.)
5'	68.3556	68.56	3.76 (m)	3,70 (m)
6'	17.1215	17.10	1.07 (d, *J*=6.35)	1.07 (d, *J*=6)

* Carbon numeration corrected established by COSY correlations and literature[11,12]

Supplementary References

1. Kaiser, H. & Keller-Schierlein, W. Structure elucidation of elaiophylin: spectroscopic studies and degradation. *Helv. Chim. Acta* **64,** 407–424 (1981).
2. Higginbotham, S. J. *et al.* Bioactivity of fungal endophytes as a function of endophyte taxonomy and the taxonomy and distribution of their host plants. *PLoS One* **8,** e73192 (2013).
3. Trager, W. & Jensen, J. B. Human malaria parasites in continuous culture. *Science* **193,** 673–5 (1976).
4. Almanza, A., Coronado, L., Tayler, N., Herrera, L. & Spadafora, C. Automated synchronization of *P. falciparum* using a temperature cycling incubator. *Curr. Trends Biotechnol. Pharm.* **5,** 1130–1133 (2011).
5. Corbett, Y. *et al.* A novel DNA-based microfluorimetric method to evaluate antimalarial drug activity. *Am. J. Trop. Med. Hyg.* **70,** 119–24 (2004).
6. Martínez-Luis, S., Gómez, J. F., Spadafora, C., Guzmán, H. M. & Gutiérrez, M. Antitrypanosomal alkaloids from the marine bacterium Bacillus pumilus. *Molecules* **17,** 11146–55 (2012).
7. Díaz-Chiguer, D. L. *et al.* In vitro and in vivo trypanocidal activity of some benzimidazole derivatives against two strains of Trypanosoma cruzi. *Acta Trop.* **122,** 108–12 (2012).
8. Bonev, B., Hooper, J. & Parisot, J. Principles of assessing bacterial susceptibility to antibiotics using the agar diffusion method. *J. Antimicrob. Chemother.* **61,** 1295–1301 (2008).
9. Ericsson, H., Tunevall, G. & Wickman, K. The paper disc method for determination of bacterial sensitivity to antibiotics: relationship between the diameter of the zone of inhibition and theminimum inhibitory concentration. *Scand. J. Clin. Lab. Invest.* **12,** 414–422 (1960).
10. Mejia, L. C. *et al.* Endophytic fungi as biocontrol agents of *Theobroma cacao* pathogens. *Biol. Control* **46,** 4–14 (2008).
11. Nair, M. G. *et al.* Gopalamicin, an antifungal macrodiolide produced by soil actinomycetes. *J. Agric. Food Chem.* **42,** 2308–2310 (1994).
12. Nakakoshi, M., Kimura, K., Nakajima, N., Yoshihama, M. & Uramoto, M. SNA-4606-1, a new member of elaiophylins with enzyme inhibition activity against testosterone 5 alpha-reductase. *J. Antibiot. (Tokyo).* **52,** 175–7 (1999).
